# Parity‐Frequency‐Space Elastic Spin Control of Wave Routing in Topological Phononic Circuits

**DOI:** 10.1002/advs.202404839

**Published:** 2024-07-31

**Authors:** Yao Huang, Chenwen Yang, Weitao Yuan, Yuxuan Zhang, Yongdong Pan, Fan Yang, Zheng Zhong, Jinfeng Zhao, Oliver B. Wright, Jie Ren

**Affiliations:** ^1^ School of Aerospace Engineering and Applied Mechanics Tongji University 100 Zhangwu Road Shanghai 200092 P. R. China; ^2^ Center for Phononics and Thermal Energy Science China‐EU Joint Lab on Nanophononics Shanghai Key Laboratory of Special Artificial Microstructure Materials and Technology School of Physics Science and Engineering Tongji University Shanghai 200092 P. R. China; ^3^ Applied Mechanics and Structure Safety Key Laboratory of Sichuan Province School of Mechanics and Aerospace Engineering Southwest Jiaotong University Chengdu Sichuan 610031 P. R. China; ^4^ School of Science Harbin Institute of Technology Shenzhen 518055 P. R. China; ^5^ Graduate School of Engineering Osaka University Yamadaoka 2‐1 Suita Osaka 565‐0871 Japan; ^6^ Hokkaido University Sapporo Hokkaido 060‐0808 Japan

**Keywords:** elastic spin, phononic circuits, topological cavities, wave routing

## Abstract

Topological phononic cavities, such as ring resonators with topological whispering gallery modes (TWGMs), offer a flexible platform for the realization of robust phononic circuits. However, the chiral mechanism governing TWGMs and their selective routing in integrated phononic circuits remain unclear. This work reveals, both experimentally and theoretically, that at a phononic topological interface, the elastic spin texture is intricately linked to, and can be explained through a knowledge of, the phonon eigenmodes inside each unit cell. Furthermore, for paired, counterpropagating TWGMs based on such interfaces in a waveguide resonator, this study demonstrates that the elastic spin exhibits locking at discrete frequencies. Backed up by theory, experiments on kHz TWGMs in thin honeycomb‐lattice aluminum plates bored with clover‐leaf shaped holes show that together with this spin‐texture related angular‐momentum locking mechanism at a single topological interface, there are triplicate parity‐frequency‐space selective wave routing mechanisms. In the future, these mechanisms can be harnessed for the versatile manipulation of elastic‐spin based routing in phononic topological insulators.

## Graphical abstract

This study reveals that the elastic spin texture is intricately linked to the phonon eigenmodes inside each unit cell at a phononic topological interface. Furthermore, for paired counterpropagating TWGMs based on such interfaces in a waveguide resonator, the elastic spin exhibits locking at discrete frequencies, and there are triplicate parity‐frequency‐space selective wave routing mechanisms.

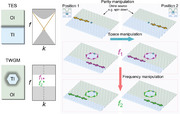

## 1 Introduction

Topological insulators (TIs) composed of lattice structures have allowed the creation of exotic, robust and chiral topological edge states (TESs) for electromagnetic,^[^ ^1^, ^2^, ^3^, ^4^, ^5^ ^]^ acoustic,^[^ ^6^, ^7^, ^8^ ^]^ and elastic waves^[^ ^9^, ^10^, ^11^, ^12^, ^13^ ^]^—bosonic fields that possess integer spin quantum number. Such progress has recently been extended to unique topological‐state configurations, including topological whispering gallery modes (TWGMs) in waveguide resonators,^[^ ^14^, ^15^, ^16^, ^17^ ^]^ corner states^[^ ^9^, ^18^, ^19^, ^20^ ^]^ in 0D (zero‐dimensional) closed interfaces,^[^ ^1^, ^10^, ^21^ ^]^ or high‐order states in 3D periodic structures.^[^ ^22^, ^23^, ^24^, ^25^, ^26^ ^]^ TWGMs exhibit topological protection and pseudospin‐locking,^[^ ^10^, ^15^ ^]^ properties which are described by subtler physics than conventional whispering gallery modes (WGMs).^[^ ^27^, ^28^ ^]^ Such features have promising applications in advanced information processing, enabling the creation of arbitrarily shaped waveguides^[^ ^14^, ^29^ ^]^ and imaging devices,^[^ ^30^ ^]^ for example.

However, the sublattice‐scale configuration within typical interfaces of TIs supporting TESs or TWGMs can be extremely intricate owing to the use of structures with broken space‐inversion symmetry,^[^ ^10^, ^31^, ^32^, ^33^, ^34^ ^]^ hindering the full manipulation of classical bosonic wave fields. The pseudospin state^[^ ^1^, ^3^, ^4^, ^10^, ^35^ ^]^—a dimensionless entity usually formed through a combination of different states in a lattice system—represents the effective polarization related to the clockwise or counterclockwise energy flux vector of a TWGM. However, the pseudospin does not suffice to completely describe the physics of a TWGM. Rather, real physical quantities must be considered to fully describe them,^[^ ^10^, ^36^, ^37^ ^]^ quantities that have the potential to be tailored by multiply‐triggered, namely, parity‐, frequency‐ and spatially‐controlled, source manipulation. “Parity control” here refers to chiral control, which is achieved, for example, by changing the source polarization to reverse the wave direction. However, this control has not been carried out effectively to date. Moreover, the field of acoustic TWGMs remains fractured and incomplete, and an overreaching physical understanding of the underlying concepts governing such control of the unidirectional waveguiding remains elusive, as summarized here: 1) For acoustic chiral (i.e., parity) control, there have been numerous studies^[^ ^35^, ^38^, ^39^ ^]^ involving the pseudospin degree of freedom in both quantum spin Hall effect (QSHE) and quantum valley Hall effect (QVHE) phononic crystals, for both TESs and TWGMs. In QSHE‐like phononic crystals, the pseudospin is usually represented by an overall acoustic polarization within the unit cell rather than by a local polarization, and spatial control is hard to achieve. 2) Previous work on QVHE‐like phononic crystals^[^ ^40^ ^]^ has demonstrated that spatial control of one‐way transmission can be enabled by changing the position and polarization direction of a chiral acoustic source; chiral and spatial control were implemented together on the same QVHE system, but only for TESs not for TWGMs. 3) Previous work on QSHE phononic crystals^[^ ^41^ ^]^ has demonstrated opposite energy flow of paired TWGMs at different frequencies by considerations of pseudospin, but no spin source was used. From these observations, one can thus conclude that triplicate parity‐frequency‐space control for a given structure has yet to be achieved. Parity‐frequency‐space control refers to adjusting the polarization (and therefore the chirality), the frequency, and the spatial position of the excitation source for the control of wave propagation.

The spin angular momentum (AM) is an inherently local quantity that offers a new dimension for the description of transverse electromagnetic waves,^[^ ^42^, ^43^, ^44^, ^45^ ^]^ longitudinal acoustic waves,^[^ ^46^, ^47^, ^48^ ^]^ and complex elastic wave polarizations.^[^ ^49^, ^50^, ^51^, ^52^, ^53^ ^]^ For classical waves, spin was widely investigated in electromagnetic wave systems, but it was not until recently that the elastic spin^[^ ^49^ ^]^ came into focus in acoustic wave systems. This is partly owing to the common misconception that only circularly polarized waves can exhibit non‐zero spin.^[^ ^42^ ^]^ Another reason is related to the topological properties related to spin AM. Electromagnetic waves can exhibit QSHE‐like behavior with a non‐zero spin Chern number, but it is challenging to derive spin Chern numbers for complex modes in acoustic systems. In fact, elastic spin is crucial for a full interpretation of acoustic wave propagation in both homogenous materials and inhomogeneous materials such as phononic crystals (PhCs) or metamaterials, for example, for waves such as bulk plane waves, superpositions of bulk waves, interface waves without topological properties or interface waves with with topological properties.

Because the subject of elastic spin is in its infancy, many related studies remain unexplored. In the acoustics of homogenous materials, for example, although the spin of Lamb waves and Rayleigh waves in semi‐infinite bulk materials have been reported,^[^ ^50^ ^]^ the characteristics of the spin AM of such waves in the depth direction have not been investigated. In addition, the spin AM for waves in rod structures, which host bending, longitudinal, and torsional modes, has not been properly investigated.^[^ ^54^ ^]^ These propagational modes exhibit elliptically‐polarized displacement fields, spin AM, and topological properties. For the case of inhomogeneous materials such as PhCs or metamaterials, it is not widely understood that elastic waves therein exhibit intrinsic spin AM. In contrast, numerous studies have been devoted to constructing pseudospin states at interfaces to achieve QSHE‐like unidirectional transport in such materials. With the revelation that elastic waves do indeed exhibit spin AM in simple structures such as plates and semi‐infinite solids, studies of valley PhCs emerged,^[^ ^40^ ^]^ allowing the possibility of spin control in topological metamaterials in addition to such control at non‐topological interfaces.

When particles undergo regular rotational motions in a dynamical classical wave system, the spin AM describes the rotation of the displacement vector at point in space. In an elastic system, acoustic waves traveling in different directions can be excited by elastic loads, namely “spin sources”, of different polarization. Elastic energy flow represents the energy transfer accompanying the propagation of acoustic waves in a medium, and reveals the path of wave propagation; it can therefore be used to confirm wave locking mechanisms in phononic circuits. There is a close correlation between the elastic energy flux vector (the acoustic Poynting vector) and the elastic spin,^[^ ^52^ ^]^ but to date the expected locking relationship between the two has not been demonstrated.

The problem of incorporating the concept of spin AM in TWGMs depends on the detailed structure of the lattice. In acoustics, for the case of valley PhC plates, for example, the eigenfrequencies of paired TWGMs are so similar that they cannot be distinguished in experimental settings.^[^ ^16^, ^17^ ^]^ In contrast, this frequency difference for PhC plates with *p*/*d* symmetry inversion properties (equivalently, a system mimicking the QSHE),^[^ ^10^, ^41^ ^]^ where *p* and *d* refer to orbital‐like acoustic eigenstates, is sufficiently large to be experimentally accessible. The term “*p*/*d* symmetry inversion” refers to the inversion of bands where the *p* and *d* modes are located. This provides a potential route to fully elucidating the physics of topological TWGMs. However, even in this case there remain significant challenges that have impeded progress.

First, the elastic spin distribution, crucial for understanding the physical origins of acoustic topological waveguiding inside a TES, even on a 1D interface, remains unknown. Considering the existing methods for obtaining (QSHE‐like) *p*/*d*‐like symmetry inversion at determinate^[^ ^4^, ^7^, ^55^ ^]^ or accidental^[^ ^10^, ^35^ ^]^ four‐fold Dirac cones in k‐space, the hitherto proposed pseudospin‐dependent energy flow fails to fully describe the underlying physics: in contrast, an elastic‐spin based theoretical analysis is essential for the proper understanding of spin‐momentum locking in TES.

Second, simulations and experiments of elastic spin at TWGM topological interfaces have not been carried out using previously developed subwavelength and tunable chiral sources.^[^ ^56^ ^]^ Such research has moreover been impeded by closely spaced paired TWGM frequencies.

Third, a detailed analysis of the directed acoustic energy flux in TWGMs has not been forwarded. Indeed, previous related work^[^ ^10^, ^37^ ^]^ did not succeed in demonstrating spin‐momentum locking, which has hindered an in‐depth understanding of the wave routing in such systems.

These hurdles have prevented the proper implementation of elastic‐spin based devices, which have a huge potential for application to chiral emitters, arbitrary shaped waveguides and imaging devices. The gates to significantly advance design capabilities for devices with arbitrary chiral transport have therefore remained tightly shut.

In this paper, we remedy this situation by revealing spin‐momentum locking in TESs and TWGMs by means of experiment, theory and simulations. To this end, we make use of thin aluminum honeycomb‐lattice PhC plates exhibiting *p*/*d* symmetry inversion, bored with clover‐leaf shaped holes, and elucidate the elastic spin distribution arising from the hybridization of both *p*‐ and *d‐*like waves at kHz frequencies. The acoustic displacement vector is first measured on a straight topological interface, confirming the presence of spin‐momentum locking in a TES. Then, by harnessing in‐plane dual‐phase subwavelength spin sources, we demonstrate unidirectional routing on this interface. Furthermore, we architect a waveguide‐resonator structure with this design methodology that contains both paired TWGMs and TESs, thereby demonstrating wave propagation inversion by modifying not only the spin source chirality, but, crucially and uniquely, also its spatial position and frequency. Moreover, we also reveal elastic spin‐momentum and pseudospin‐momentum locking, as well as the chirality of energy flux vector field in a waveguide‐resonator phononic circuit for the first time.

## 2 Results

### 2.1 Overall Research Focus

**Figure 1:**
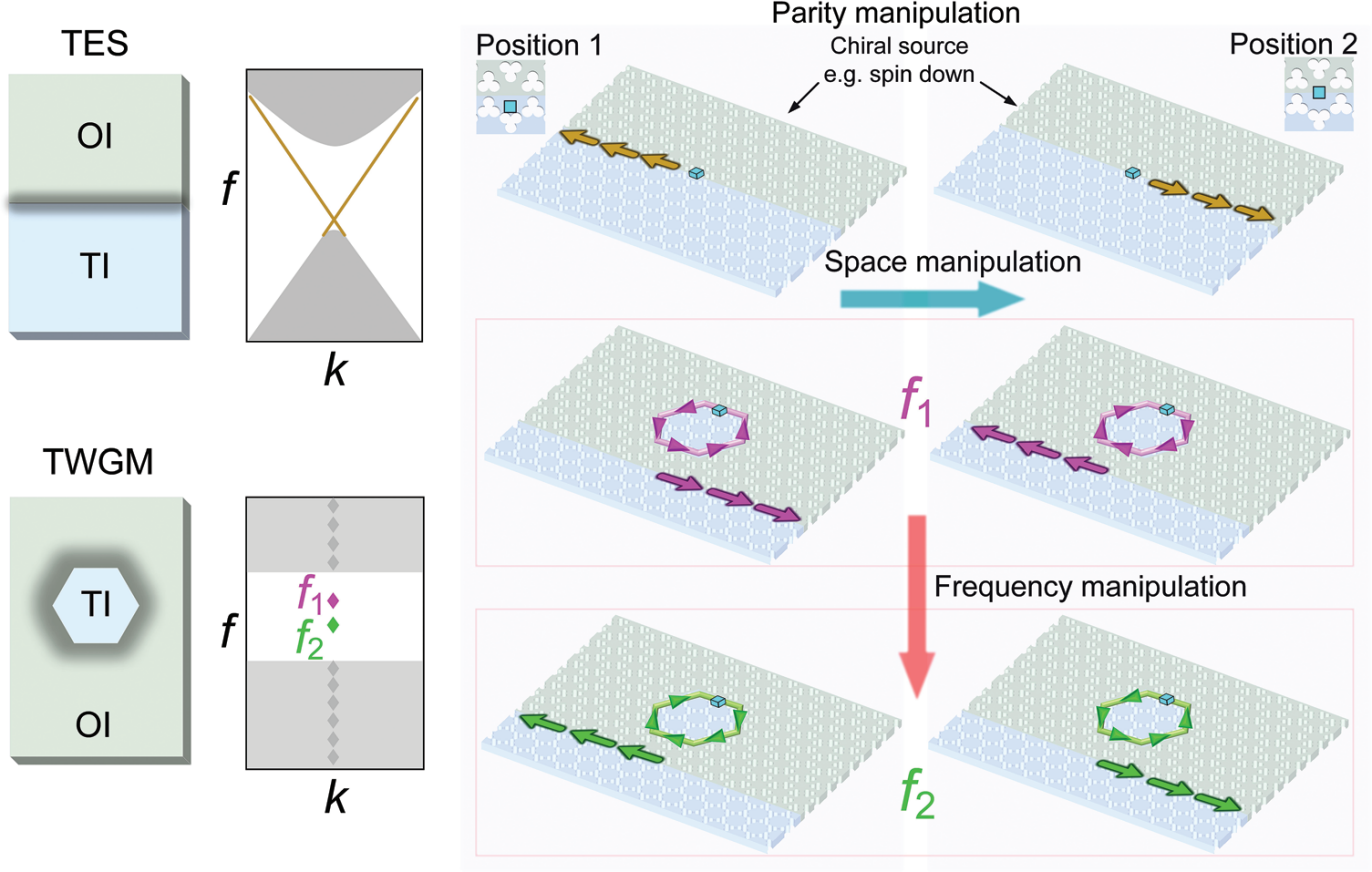
Schematic of samples and dispersion for parity‐frequency‐space spin control of wave routing. a) PhC and dispersion for the TES (upper panels) and TWGM (lower panels). b,c) Multiply‐triggered spin control of wave routing by varying the source location or excitation frequency with a chiral source (e.g., a spin‐down spin source), for straight TESs (upper panels) and waveguide‐resonator structures (middle and lower panels). The source location in (b,c) for both a TES (upper panels) and a TWGM (middle and lower panels) are denoted as positions 1 and 2, respectively, and the frequencies of paired TWGMs are denoted by *f*_1_ (middle panels) and *f*_2_ (lower panels).

We first summarize the overall focus of our study on multiply‐triggered selective TWGM wave routing by reference in **Figure** **1** to schematics of the samples used and outlines of their acoustic dispersion. We constructed a straight TES in a PhC, as shown on the left of the upper panel of Figure 1a, whose dispersion (shown on the right) is continuous over the bandgap of the antisymmetric plate wave of the ordinary insulator (OI) and the topological insulator (TI). Two opposite wave routes are enabled in this honeycomb‐lattice PhC, constructed from a thin aluminum plate bored with clover‐leaf shaped holes^[^ ^57^, ^58^, ^59^, ^60^ ^]^ (see inset of Figure 1b and below for details), by implementing a single spin‐down acoustic source at two different positions, as shown by the blue cubes in the upper panels of Figure 1b,c. To investigate confined modes, we constructed a hexagonal‐resonator phononic cavity based on this general architecture, as shown on the left of the lower panel in Figure 1a, making use of the same spin source. In contrast to the case for open waveguides supporting 1D TES, the hexagonal cavity features a closed circuit supporting 0D modes (shown on the right), including the TWGMs. Thus, the TWGMs frequencies are discrete and exhibit a relatively weak dependence on wave number *k*. Owing to the choice of circumference of this topological whispering gallery (TWG), there exist only one pair of eigenfrequencies, *f*_1_ and *f*_2_, within the bandgap of the antisymmetric plate waves of the OIs and TIs. We also constructed a TES‐TWGM coupling structure incorporating in addition a straight waveguide to explore the mechanism of acoustic wave locking. As with the TES, the same spin source is used at different positions with the same excitation frequency to achieve opposite wave routing, as shown in the middle and or lower panels in Figure 1b,c. Opposite routing at frequencies *f*_1_ and *f*_2_ is achieved at fixed position and chirality. We now turn to the details of our proposed structures.

### 2.2 Mapping Elastic Spin Over a TES in a PhC Plate with *p/d* Symmetry Inversion

**Figure 2:**
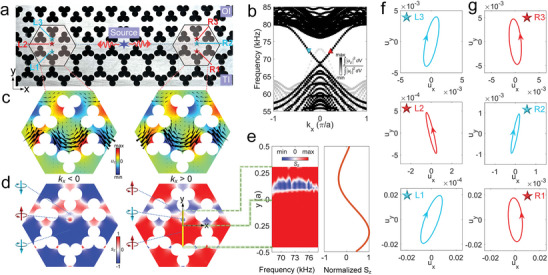
Elastic spin in a phononic topological edge state. a) Photograph of the constructed linear topological interface comprised of ordinary insulator (OI) and topological insulator (TI) regions. A point source generates left‐ and right‐directed acoustic TES at the interface, within the bandgap of antisymmetric plate waves. b) Simulated dispersion of the supercell comprised of OI and TI. The color scale indicates the ratio of *u_z_* to the total displacement *u_t_* in the supercell. The TES is dominated by the component *u_z_*. A tiny bandgap exists within the frequency range of the TES (see Figure S2, Supporting Information). c) Simulated asymmetric distribution of *u_z_* versus coordinate *y* at the points represented by the blue and red triangles in the dispersion relation (b). Opposite pseudospin states are specified by the in‐plane mechanical energy flux vector (black arrows) on the interface (upper surface of plate) at 72 kHz. See Video S1 (Supporting Information). d) Simulated distribution of *s_z_* at the interface (upper surface of plate) under the same condition as in (c). The red/cyan rings with up/down arrows represent spin‐up/down states. Normalized *s_z_* is indicative of the elliptical displacement polarization in the *x*‐*y* plane, and circular polarization occurs when |*s_z_* | = 1. See Video S2 (Supporting Information). e) Simulated elastic spin density *S_z_* (left panel) versus frequency, and theoretical *S_z_* (normalized to the maximum *S_z_*) at 72 kHz (right panel) along the *y*‐axis, as denoted by the yellow line in (d). f,g) Measured displacement polarization at position L1‐3 for left‐directed waves, and at position R1‐3 for right‐directed waves, when *f* = 73 kHz.

The PhC slabs are fabricated by drilling a hexagonal honeycomb lattice of clover‐leaf shaped holes on aluminum plates of thickness *h* = 9.2 mm and lattice constant *a* = 25 mm, as shown **Figure** **2**a (see Figure S1, Supporting Information, and in Experimental Section for the details). The topological phase of the PhC is spatially toggled with band inversion between the *p_x_* (*p_y_*) and $d_{x^{2}-y^{2}}$ (*d_xy_*) modes by adjusting the rotation angle *θ* of the clover‐leaf holes. Juxtaposing an OI phase (*θ* = 0°) and a TI phase (*θ* = 60°) engenders two TESs, as implied by the band structure in Figure 2b, including rightward‐ and leftward‐directed modes along the interface within the bandgap 68–75.4 kHz of the antisymmetric plate modes. By choosing a frequency of 72 kHz (corresponding to a wavelength of 23.5 mm and sound velocity 1692 m s^−1^) in the middle of the frequency band of the TES as an example, the simulations of the in‐plane mechanical energy flux vector of Figure 2c (see Methods for details) clearly show the presence of opposite pseudospin states (see theoretical expressions in Note S4, Supporting Information), represented by an energy‐flow vortex (depicted as black arrows) in the hexagonal unit cell. Figure 2d shows the simulated distribution of the normalized elastic spin density *s_z_* ^[^ ^56^ ^]^ (along the *z*‐axis), defined by *s_z_* = Im(*u_x_* ^*^ *u_y_*‐*u_x_u_y_* ^*^)/(|*u_x_* |^2^+|*u_y_* |^2^), describing the ellipticity of the in‐plane displacement field, which reveals the “spin up/down/up” spin distribution (for *k_x_* > 0) along the central symmetry line (yellow line) in the unit cell. This distribution of *s_z_* differs from the simple rotational handedness of the in‐plane energy flux vector, and breaks the previously assumed criterion that the TES features only one chirality in a unit cell for a given propagation direction:^[^ ^3^ ^]^ that is, it has been assumed that a PhC with (QHSE‐like) *p*/*d* symmetry inversion will generally have only a single chirality arising solely from the energy flow related to the pseudospin within a unit cell (where only one energy flow vortex exists). This conclusion also differs from that of a preceding study of the elastic spin in a valley‐PhC plate,^[^ ^40^ ^]^ in which case the energy flow vortex only locks to one sign of the elastic spin.

Figure 2e (left panel) shows the simulated frequency‐dependent 1D distribution of the elastic spin density *S_z_*, defined by *S_z_* = (*ρω*/2)Im(*u_x_* ^*^ *u_y_*‐*u_x_u_y_* ^*^),^[^ ^40^ ^]^ representing the real spin angular momentum associated with the elastic vibration for this PhC structure, exhibiting a broadband “spin up/down/up” (*k_x_* > 0) spin distribution (corresponding to the yellow line, i.e., for *x* = 0, in Figure 2d). To shed light on the physical origin of *S_z_*, we return to the locally asymmetric distribution of *u_z_* of the TES, as shown in Figure 2c, resulting from the hybridization of the *p_y_* and *d_xy_* modes.^[^ ^10^ ^]^ This hybridization can be validated by extracting *u_z_* even on a small portion of the interface, e.g., at 72 kHz, and separating the displacement into *p_y_* and *d_xy_* modes through mode decomposition. (See also Figure S3, Supporting Information). The theoretical displacement distribution of *u_z_* for the *p_y_* and *d_xy_* modes is assumed to be given by *u_p_* = sin(*πy*/*a*)[1 + 0.25cos(*πx*/*a*)] and *u_d_* = sin(*πy*/*a*)sin(*πx*/*a*) for the area ‐*a*/2 < *x* <*a*/2 and ‐*a*/2 < *y* < *a*/2. The TES is described by *u_z_* = [*ζe* ^i^ *^φ^u_p_* (*x*,*y*) + *u_d_* (*x*,*y*)]*R*(*y*), for which the real value *ζ* ^[^ ^36^ ^]^ is related to the wave number, *e* ^i^ *^φ^* is a phase factor that accounts for the difference between the *p_y_* and *d_xy_* modes, and the decaying function *R*(*y*) is fitted in the form *R*(*y*) = exp(−0.62|*y|*/*a*) from the experimental data. (See also Figure S4, Supporting Information). This theoretical solution for *u_z_* combined with thin‐plate theory enables one derive the elastic spin density *S_z_* = (*ρω*/2)Im(*u_x_* ^*^ *u_y_*‐*u_x_u_y_* ^*^)^[^ ^49^ ^]^ at the interface (see Note S4, Supporting Information, for details).

Figure 2e (right panel) shows the corresponding theoretically calculated 1D distribution of *S_z_* (i.e, *S_z_* normalized to its maximum value) at 72 kHz (for *k_x_* > 0). The theoretical “spin up/down/up” 1D spin distribution (for *k_x_* > 0) in each hexagonal unit cell on the OI‐TI interface is consistent with the simulated 1D profile (the left panel of Figure 2e), albeit there being a slight frequency shift owing to the decaying function *R*(*y*) in the theoretical *S_z_* being obtained through fitting to experiment (see Note S4, Supporting Information, for details). For the case *k_x_* < 0 (not shown), the sign of *S_z_* in Figure 2e is reversed as expected in both simulation and theory. Owing to the decay in the function *R*(*y*), the simulated *S_z_* decays quickly along the transverse direction, exhibiting a relatively small value even in the neighboring row (see Figure S5, Supporting Information). However, this transverse decay is not mirrored by the spin *s_z_* because of its normalization.

For the experimental observation of elastic spin, the PhC is fabricated by machining from a single aluminum plate. A PZT‐5H disc (radius 2 mm and thickness 0.5 mm) is attached to the middle of the topological interface to excite both left‐ and right‐directed waves (red arrows in Figure 2a). Subwavelength cubes (see below for details) are fixed at the positions L1‐3 and R1‐3 to measure constant‐frequency local elastic spin for both left‐ and right‐directed waves, respectively. Displacements perpendicular to the adjacent side surfaces of each source are recorded to track the temporal evolution of both *u_x_* and *u_y_* at the selected position. The amplitude and phase of *u_x_* and *u_y_* are then extracted at a given frequency, as well as their temporally‐resolved profiles (see Figure S6, Supporting Information). Figure 2f shows the 73‐kHz clockwise polarized displacement at L1 and L3 and anticlockwise polarized displacement at L2. Figure 2g shows that the polarizations at R1‐3 for rightward‐traveling waves are inverted compared the case of L1‐3 in Figure 2f for leftward‐traveling waves. The measured polarization directions in Figure 2f,g fit well with the simulated “spin down/up/down” elastic spin profiles (*k_x_* < 0) and with those for “spin up/down/up” (*k_x_* > 0) in Figure 2d.

### 2.3 Parity‐Frequency‐Space Spin Control of Wave Routing in a TES

**Figure 3:**
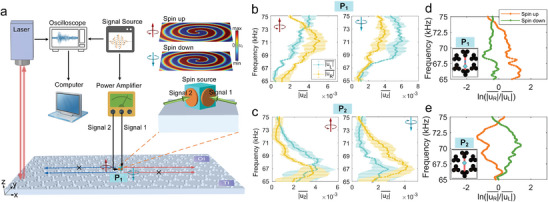
Experimental spin‐dependent unidirectional transmission. a) Schematic of the experimental setup. The circularly‐polarized spin‐up/down spin source (middle right panel) is placed at the center of an OI‐TI interface to excite unidirectional waves. Simulated spiral displacement fields *u_z_* (in *x*‐*y* plane) resulting from such spin sources (the same size as those in experiment) positioned on an undrilled aluminum plate are plotted in the upper‐right panel for square regions of side 4.5*a*. Elastic waves are observed by detecting *u_z_* on the *x*‐*y* surface with a laser Doppler vibrometer. b,c) Averaged |*u_z_* | measured on the right (left) side of the spin source, denoted as $\bar{|u_{R}|}$ ($\bar{|u_{L}|}$). Spin‐up/down sources are placed at points P_1_ and P_2_ (see light‐blue squares in (d,e)), with central excitation frequency *f_c_* = 72 kHz. d,e) Frequency‐dependent difference of |*u_z_* | averaged over right‐ and left‐directed waves, denoted by $\ln(\bar{|u_{R}|}/\bar{|u_{L}|})$ under conditions of spin‐up (orange line) or spin‐down (green line) source excitation.

We now turn to the topic of selective wave routing. Locking between local spin and wave momentum (or wave vector) lays the foundation for source‐position spatially‐triggered spin‐momentum coupling in TESs. Here we develop a new subwavelength in‐plane dual‐phase elastic spin source tailored to our requirement for directional excitation; it consists of two PZT‐5H discs (orange disks shown in **Figure** **3**a of thickness 0.5 mm and radius 2 mm) bonded with epoxy adhesive on both sides of an aluminum parallelepiped (of dimensions 4 × 4 × 3 mm^3^), which is then similarly bonded to the OI‐TI interface. Two tone bursts (signals 1 and 2) are applied to the PZT discs with the same amplitude but having a phase difference of ±π/2 with respect to the central frequency of the tone bursts, leading to a rotational vibration of the transducer cubes about a vertical axis and of the adjacent regions of the aluminum plate. This elastic spin source contrasts with those we previously developed, whereby out‐of‐plane generation is used.^[^ ^40^, ^56^ ^]^ Here we directly generate *x*‐ and *y*‐directed displacements with an in‐plane dual‐phase spin source, yielding a pure and circularly‐polarized *S_z_* component as required by our chosen structures. Our spin source has the advantage of not coupling to resonances of the aluminum parallelepiped in the frequency range of interest. The upper‐right panel in Figure 3a depicts the spin‐up/down elastic spin source together with the simulated anticlockwise/clockwise displacement field component *u_z_* at the surface of an undrilled aluminum plate.

Two positions in the PhC slab are then selected with opposite *S_z_*, i.e., points P_1_ (for *k_x_* > 0, *S_z_* > 0; *k_x_* < 0, *S_z_* < 0) and P_2_ (for *k_x_* > 0, *S_z_* < 0; *k_x_* < 0, *S_z_* > 0) that correspond to the point pairs (R1, L1) and (R2, L2) in Figure 2a, respectively. In this context, the normalized quantity *s_z_* at P_1_ or P_2_ takes a value close to ±1, i.e., indicative of circular polarization, so that a circularly‐polarized chiral source is a suitable choice at these points. Spin up/down spin sources are driven with a five‐cycled tone burst pulse centered at 72 kHz. By first locating the source at P_1_, we record *u_z_* along the OI‐TI interface for both right‐ and left‐traveling waves by means of a laser ultrasonic imaging technique.^[^ ^50^ ^]^ The results for *u_z_* recorded along the OI‐TI interface, as well as its normalized 2D Fourier transform (FT) modulus (see Figure S7, Supporting Information) testify to the success in generating a TES. We extract the average amplitude of *u_z_* in selected regions along the interface for both right‐directed ($\bar{|u_{R}|}$) and left‐directed ($\bar{|u_{L}|}$) waves. As shown by the experimental frequency versus $\bar{|u_{z}|}$ plots of Figure 3b, we observe at point P_1_ asymmetric rightward propagation ($\bar{|u_{R}|}$ > $\bar{|u_{L}|}$) over the range 65–73 kHz with a spin‐up source (see the left panel) and leftward propagation ($\bar{|u_{R}|}$ < $\bar{|u_{L}|}$) over the range 68–75 kHz with a spin‐down source (see the right panel), in good agreement with the values of *S_z_* at P_1_ for rightward/leftward‐traveling waves. As shown by similar plots in Figure 3c, at point P_2_ the difference between $\bar{|u_{R}|}$ and $\bar{|u_{L}|}$ is clearly visible over a wide frequency range, i.e., for $\bar{|u_{R}|}$ < $\bar{|u_{L}|}$ (respectively, $\bar{|u_{R}|}$ > $\bar{|u_{L}|}$) over the range 67–74 kHz, by using a spin‐up (spin‐down) source, which is the opposite case to the results in Figure 3b.

Figure 3d,e shows experimental plots for the frequency versus the ratio $\ln(\bar{|u_{R}|}/\bar{|u_{L}|})$ at selected source positions. In particular, in Figure 3d at point P_1_, the positive/negative value of $\ln(\bar{|u_{R}|}/\bar{|u_{L}|})$ represents rightward/leftward wave routing, according to generation by a spin up/down spin source. Conversely, as shown in Figure 3e, the right/left‐directed wave at point P_2_ is excited by the spin down/up spin source, owing to the opposite value of *S_z_* at the points P_1_ and P_2_. Strikingly, as shown in Figure 3e, the ratio $\ln(\bar{|u_{R}|}/\bar{|u_{L}|})$ can reach ≈1.5 within the frequency range of the TES, which differs from the results in Figure 3d. This is due to experimental errors in the position and size of the fabricated spin source. The inversion of the ratio $\ln(\bar{|u_{R}|}/\bar{|u_{L}|})$ near 75 kHz is thought to be caused by the imperfect installation of the spin source, sample parameter fluctuations over each unit cell, or interference from bulk modes. Relevant simulated results show a broadband spin‐momentum coupling similar to that seen in experiment. (See Figure S8, Supporting Information).

### 2.4 Parity‐Frequency‐Space Elastic Spin Control of Wave Routing

**Figure 4:**
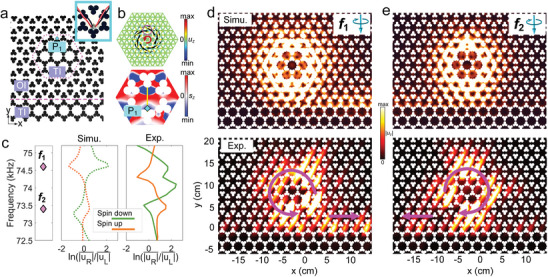
One‐way routing in a waveguide resonator at point P_1_. a) Experimental waveguide‐resonator structure incorporating a straight OI‐TI waveguide (purple dashed line) coupled to the hexagonal ring resonator (purple dotted line), comprised of a TI embedded in an OI. A spin source (blue square near the upper interface and enlarged in the inset) is used to selectively excite one‐way propagating waves. b) Simulated distribution of *u_z_* for a TWGM at the eigenfrequency *f*_1_ = 74.6 kHz (upper panel), and the simulated distribution of *s_z_* (lower panel) over a zoomed‐in view of a unit cell (purple solid line) at the upper edge of the resonator. c) Simulated eigenfrequencies (left panel) of the hexagonal supercell (upper panel of (b)). Plots of frequency versus $\ln(\bar{|u_{R}|}/\bar{|u_{L}|})$ for simulation (middle panel) and experiment (right panel), where $\bar{|u_{R}|}$ ($\bar{|u_{L}|}$) is the average of |*u_z_* | measured along the straight OI‐TI waveguide on the right (left) side of the TWG. Spin‐up (orange line) or spin‐down (green line) sources are located at point P_1_ on the upper edge of the waveguide resonator in (a). d) Simulated (upper panel at *f*_1_ = 74.6 kHz) and experimental (lower panel at 74.4 kHz) distributions of |*u_z_* |. e) Simulated (upper panel) and experimental displacement (lower panel) distributions for |*u_z_* | together with the in‐plane energy flux vector (purple arrows), both at *f*_2_ = 73.4 kHz. A spin‐down source is used for both (d,e).

Having theoretically and experimentally confirmed the spin‐momentum coupling of TESs in a system with a linear OI‐TI interface, we extend our investigations to waveguide resonators. Such resonators have key applications in coherent energy storage, filtering and wave sources. Considerable effort has been dedicated to experimentally achieving one‐way transmission at specific single frequencies in waveguide resonators. This is due to the persistent challenge of distinguishing between paired TWGMs. Here we apply the concepts of elastic spin to such structures to achieve multifunctional wave manipulation, namely parity‐frequency‐space spin control on a single structure. We focus on the particular waveguide‐resonator structure of **Figure** **4**a, based on the same PhC as used for the linear interface.

Figure 4b (upper panel) shows the supercell of the hexagonal ring resonator between the TI (inside) and the OI (outside). Figure 4c (left panel) shows the eigenfrequencies of the pair of TWGMs at *f*_1_ = 74.6 kHz and *f*_2_ = 73.4 kHz within the bandgap of antisymmetric plate waves. Figure 4b (upper panel) shows the calculated *u_z_* at *f*_1_ = 74.6 kHz, for the mode confined to the ring path of resonator, together with the anticlockwise in‐plane energy flux vector along the ring path. For comparison, *s_z_* in a selected unit cell (purple solid line) at the upper edge of the ring resonator is shown by lower panel of Figure 4b. The “spin down/up/down” feature is clearly visible along the central vertical line. At *f*_2_ = 73.4 kHz, the calculated in‐plane energy flux direction and *s_z_* are opposite to the results at *f*_1_ (see Figure S9, Supporting Information).

On putting a chiral source at P_1_, we show in Figure 4c (middle panel) the calculated profiles of the ratio $\ln(\bar{|u_{R}|}/\bar{|u_{L}|})$, with the peak and dip showing a good correspondence with the eigenfrequencies of the TWGMs (at *f*_1_ and *f*_2_). Around *f*_1_ = 74.6 kHz, the leftward (rightward) wave with negative (positive) $\ln(\bar{|u_{R}|}/\bar{|u_{L}|})$ is excited by the spin‐up (spin‐down) source. At *f*_2_ = 73.4 kHz, the results show the opposite behavior in comparison to those at *f*_1_. For spin‐up and spin‐down sources at point P_1_, the experimental 2D‐FFT spectrum of *u_z_* along the straight OI‐TI waveguide (purple dashed line in Figure 4a) is discussed in Note S7 (Supporting Information) (see also Figure S11, Supporting Information). Overall, the experimental curves (right panel in Figure 4c) fit well with the simulations, except for a slight frequency shift of the peak or dip position at *f*_1_ in experiment. These we believe are caused by small deviations in the simulated parameters, such as hole size and elastic constant, from those in experiment.

Figure 4d (upper panel) shows a simulated 2D map of |*u_z_* | at *f*_1_ = 74.6 kHz corresponding to the use of a spin‐down source at point P_1_ to produce anticlockwise wave circulation around the ring path of the resonator at *f*_1_, together with the right‐directed wave on the straight OI‐TI waveguide. The corresponding experimental results are shown in the lower panel. Figure 4e (upper and lower panels) shows the corresponding simulated and experimental 2D maps of |*u_z_* | at *f*_2_ = 73.4 kHz, exhibiting the opposite behavior. Likewise, when using the spin‐up source at P_1_ with the frequency set at either *f*_1_ or *f*_2_, both the simulated and experimental wave circulation are reversed compared to the spin‐down case (see Figure S9, Supporting Information). In contrast to the simulation, the light spots around the ring in experiment in Figure 4d,e are not evenly distributed, some diagonal stripes occurring in certain areas. The origin of this phenomenon is not clear but may be linked to the finite spatial resolution of the spatial scanning (see Experimental Section).

The coupling between the waveguide resonator and the straight OI‐TI interface can be understood through a TI‐OI‐TI supercell interface (see Figure S12, Supporting Information), even without a complex theory of elastic spin AM, because spin AM only provides local information on the dynamics. Nevertheless, at *f*_1_ or *f*_2_, the local spin *S_z_* of the TWGM locks to the wave direction (implying a corresponding directional energy flux along the ring path of the resonator); *S_z_* at P_1_ or P_2_ are both frequency dependent (opposite at *f*_1_ and *f*_2_) and position dependent (opposite at P_1_ and P_2_), which strongly contrasts with the case of a TES.

Our ring resonator supports other TWGMs, e.g., at two frequencies *f*_3_ and *f*_4_ in the bulk band of the asymmetric plate wave (as described in detail in Note S8, Supporting Information—see also Figures S14 and S15, Supporting Information). However, because of coupling to bulk waves, the distribution of *S_z_* is reshaped at these frequencies, and the unidirectional wave routing at *f*_3_ or *f*_4_ is not as efficient that evidenced at *f*_1_ or *f*_2_.

## 3 Discussion

We have shown for linear interface of our QSHE‐like PhC that the unique spin distribution of “spin up/down/up” (*k_x_* > 0) or “spin down/up/down” (*k_x_* < 0) arises from the particular *p*/*d* mode hybridization of the TES. This spin distribution reflects the intrinsic spin characteristics at any spatial position, and, in particular, near a defect exhibits a perturbed spatial pattern. We have also shown that spin‐momentum locking of the TES, resulting in directional transmission, depends on this specific intrinsic spin distribution at the interface induced by the (parity‐ or spatially‐controlled) spin excitation.

A strict correspondence between spin and pseudospin exists for our linear TES, where multiple spin signs (of different polarization—see Figure 2d) correspond to a single energy‐flow vortex with either a counterclockwise or clockwise polarization in Figure 2c. The counterclockwise energy flow locks to the spin up/down/up configuration in the unit cell. By incorporating the in‐plane displacement represented by the pseudospin into the equation for elastic spin, one can conclude that both spin and pseudospin are locked to the wave direction of the TES. In terms of spatial control, the roles of spin and pseudospin are different: as shown in Figure 2, if one aims for rightward directional transmission, controlling the pseudospin‐related energy flow (Figure 2c) requires a slightly larger elliptical polarization source placed in the unit cell, a method that has previously been reported. In contrast, we have demonstrated a completely different and more flexible control mechanism: namely, spin‐related energy flow (Figure 2d) can be achieved by placing differently polarized spin sources at different spatial positions within the same unit cell.

Concerning the ring cavity in our QSHE‐like PhC, the use of a small enough cavity allowed us to demonstrate by simulation and experiment the possibility of spin control at TWGM topological interfaces at closely spaced paired frequencies. In general, resonators encompassing smaller numbers of unit cells yield fewer eigenstates within the bandgap, and the frequency splitting is thus accentuated. Our constructed hexagonal resonator features a relatively short circumference (∼12*a*), and only a single pair of eigenstates in the bandgap, yielding a sufficiently enlarged frequency splitting of 1.2 kHz (*f*_1_ = 74.6 kHz and *f*_2_ = 73.4 kHz).

Our detailed analysis of the energy flux vector and spin‐momentum locking in such TWGMs promotes an in‐depth understanding of the parity‐frequency‐space dependent wave routing. In a perfectly circular resonator, there exist pairs of eigenstates with opposite energy flows at the same eigenfrequency. In contrast, in resonators with broken spatial symmetry, e.g., in a hexagonal TWG, the degeneracy is removed. However, the resulting split eigenmodes still correspond to the pseudospin‐related states |*q*, +〉 and |*q*, −〉, which respectively relate to counterclockwise and clockwise energy flow around the waveguide resonator.^[^ ^21^, ^36^, ^41^ ^]^ The TWGMs we investigate at *f*_1_ and *f*_2_ can be classified as a “mode 0 and 0′” pair, according to the distribution of *u_z_* described in Note S8 (Supporting Information) (see also Figure S13, Supporting Information).^[^ ^36^, ^41^ ^]^ These paired TWGMs naturally feature counterclockwise and clockwise energy flows, and, further, feature the pseudospin‐up and ‐down states of the waveguide resonator. We have demonstrated that the energy flows for these paired TWGMs are opposite because of broken spatial symmetry, as shown in Figure 4b (and in Figure S9, Supporting Information). In addition, time‐reversal symmetry allows the TWGMs to be reversed at a single frequency, i.e., from |*q*, +〉 to |*q*, −〉 at *f*_1_, or from |*q*, −〉 to |*q*, +〉 at *f*_2_. This leads to the demonstrated novel spin‐locking, frequency‐dependent and spatially‐tunable wave behaviors.

## 4 Conclusion and Outlook

To conclude, we have reported three main findings: first, we have revealed the elastic spin distribution on a linear QSHE‐like phononic‐crystal interface and shown that the elastic spin texture is intricately linked to, and can be explained through a knowledge of, the phonon eigenmodes inside each unit cell. This is demonstrated at a linear interface supporting a TES based on *p*/*d* symmetry inversion in a PhC comprised of clover‐leafed honeycomb‐lattice unit cells. We uncover the presence of spatially dependent spin‐momentum locking in such edge states, and ascribe its theoretical origin to the hybridization of *p* and *d* orbital‐like modes of the PhC plate, backed up by experimental observations of the elastic spin at representative positions. We also demonstrate broadband unidirectional wave routing of TESs, which arises thanks to the theoretically revealed locking mechanism of the local elastic spin to the target direction.

Second, we have shown by simulation and theoretical analysis that there is a strict relationship between the elastic spin and the mathematically‐constructed pseudospin: within a unit cell on the QSHE‐like linear topological interface studied, a single energy flow vortex corresponds to multiple spin signs, and results, for example, in a counterclockwise energy flow locked to a spin up/down/up configuration. This is distinct from the situation in a valley PhC,^[^ ^40^ ^]^ in which an energy flow vortex corresponds to a single spin sign and results, for example, in a counterclockwise energy flow locked only to the spin‐up state.

Third, we uncover by simulation, theory and experiment triplicate parity‐frequency‐space selective broadband wave routing mechanisms through a detailed analysis of the acoustic energy flux in TWGMs. These are excited by in‐plane dual‐phase spin sources in hexagonal phononic circuits coupled to linear topological waveguides, which are based on the same lattices as in the linear TESs investigated. We find that the spin and energy flow of paired TWGMs, closely spaced in frequency, is opposite, and that the counterclockwise/clockwise energy flow along the TWG lock to right/left‐going waves on the coupled linear waveguide.

Finally, this study not only enriches our understanding of elastic spin AM in TESs and TWGMs, but also demonstrates a versatile selective unidirectional acoustic generation scheme by use of subwavelength chiral sources. This study should also spur the investigation of spin texture in diverse materials and structures, including metamaterials, promoting the methodology of clearly distinguishing local spin and generalized pseudospin. This approach should lead to novel and tailorable on‐chip functional devices and components.^[^ ^32^, ^61^ ^]^ This parity‐frequency‐space one‐way routing of TWGMs also opens the way for the design of arbitrary‐path waveguiding,^[^ ^14^, ^62^, ^63^ ^]^ selective chiral emitters,^[^ ^61^, ^64^, ^65^ ^]^ and direction‐dependent energy splitters.^[^ ^66^ ^]^ Knock‐on effects, not limited to lattices with *p*/*d* symmetry inversion, are also foreseen for other bosonic wave fields such as in electromagnetism, with important implications in the development of new photonic^[^ ^1^, ^30^ ^]^ and electronic circuits.^[^ ^67^, ^68^ ^]^

## 5 Experimental Section

### Numerical simulations

The study makes use of the finite‐element method to calculate the phononic dispersion of the PhC plate, made of 6061‐T6 aluminum: Young's modulus 67.7 GPa, mass density 2.7 g cm^−3^ and Poisson's ratio 0.35. The dispersion of the PhC plate was computed from its unit cell (see Experimental Setup below), while varying the rotation angle *θ* of the clover‐leaf holes from 0° to 60° (see Figure S1, Supporting Information). A supercell (see Figure 2b) that contains 10 OI unit cells and 10 TI unit cells was constructed to simulate the dispersion of TESs and other modes. Apart from the dispersion relation of the supercell, one can retrieve the displacement ratio $\int {|u_{z}|}^{2}dV/\int {|u_{t}|}^{2}dV$.

To simulate the wave propagation, the study makes use of an OI parallelogram array (with its two side lengths containing 20 and 10 unit cells, respectively) above the TI parallelogram array (with the same size as the OI), within a rhombus‐shaped region with a side length of 500 mm (see Figure S8, Supporting Information) on a bare alumimum plate. Wave reflection at the boundary between the PhC plate and the bare plate was prohibited: perfectly matched layers (PMLs) were adopted at the periphery of the bare plate to prohibit such reflections. Both the point‐like sources and the subwavelength chiral sources are used in the simulations. To obtain the point‐like source, a unit force along the *z*‐axis was applied over a circular region of radius 2 mm situated at the center of the OI‐TI interface (see Figure 2a; Figure S6, Supporting Information). For the chiral sources at points P_1_ or P_2_, unit forces were applied perpendicular to two adjacent side surfaces of what was assumed to be an aluminum cube (see Figure S8, Supporting Information) fixed rigidly to the PhC plate. The average of |*u_z_* | within a length of 3*a* on the right‐hand (left‐hand) side of the wave source is used to obtain $\bar{|u_{R}|}$($\bar{|u_{L}|}$) and subsequently the ratio $\ln(\bar{|u_{R}|}/\bar{|u_{L}|})$, by averaging along the straight OI‐TI interface and far away from the spin source.

To simulate the elastic wave propagation for the wave‐resonator structure, an OI‐TI straight topological interface (30*a* = 750 mm) was constructed, with the OI above and the TI below. This hexagonal resonator has a side length of 50 mm, and its lower edge is situated √3*a* away from the straight OI‐TI interface. A waveguide‐resonator structure was obtained inside a parallelogram‐shaped PhC region (with side lengths 750 and 500 mm) enclosed by the bare aluminum plate (of radius 800 mm) and by the PML. The same chiral source as used above was placed on the upper edge of resonator at given point P_1_ or P_2_. Averages are obtained by the same method as before (see the middle panel in Figure 4c; Figure S10, Supporting Information).

### Experimental Setup

As mentioned in the Numerical Simulations section, the PhC plates were fabricated with 6061‐T6 aluminum of thickness *h* = 9.2 mm, in which the clover‐leaf holes were perforated by a Computer Numerical Control (CNC) milling machine to the following specification: lattice constant *a* = 25 mm, radius of air holes *r* = 3.25 mm, and neighboring air‐hole center distance *d* = 6.24 mm. In this way, a PhC plate within a rhombus‐shaped region (with a side length of 500 mm) was constructed on a bare parallelogram‐shaped aluminum plate (with side lengths 700 and 800 mm) to create the OI‐TI interface. The PhC plate was also constructed within a parallelogram‐shaped area (with side lengths 750 and 500 mm) on a separate parallelogram‐shaped aluminum plate (with side lengths 800 and 540 mm) to create the waveguide resonator structure. The sample was enclosed by the modeling clay Blu‐Tack, which serves a good acoustic absorbing layer.

For the acoustic imaging, a laser Doppler vibrometer (LDV, Polytec OFV 2570) was used to measure the out‐of‐plane displacement *u_z_* at any position on the surface of the PhC plate. The experimental data was averaged by a total of 128 times at each point. The study similarly recorded the displacement perpendicular to the two adjacent sides of the subwavelength transducer cube, and thereby measures the effective in‐plane displacements *u_x_* and *u_y_* (see Figure S6, Supporting Information) as well as their polarization.

The study makes use of both point‐like and chiral sources in experiment. For the former, a single PZT‐5H disk (radius 2 mm and thickness 0.5 mm) was bonded to the OI‐TI interface by epoxy adhesive. A five‐cycled tone burst of central frequency *f_c_* (≈72 kHz) from the signal generator (RIGOL DG1032z) was amplified by a power amplifier (Aigtek ATA‐2022H), and applied to the PZT disk. This pulse signal repeats every 30 ms. This point‐like source generates both left‐ and right‐directed acoustic waves.

A single PZT was used at the center of the topological interface (*x* = 0) to obtain 1) the displacement field for *p*/*d* mode separation, 2) the transverse profile of the normalized |*u_z_* |, and 3) the displacement polarization at a typical position. Regarding the experimental characterization of the *p*/*d* mode separation (see Figure S3, Supporting Information), a square area is selected on the OI‐TI interface, covering *x* = −5 to 5 mm and *y* = −7.5 to 7.5 mm. The displacement *u_z_* is recorded at 1 mm intervals along both the *x*‐ and *y*‐axes. The study can thereby retrieve the time‐revolved distribution of *u_z_* (namely, matrix *E*, a snapshot of the matrix of *u_z_* at a given frequency—see Figure S3, Supporting Information) in the above‐defined area at a chosen target frequency (from 70 to 75 kHz). *E* was chosen at certain moments to be representative for the experimental separation of *d_xy_* and *p_y_* by the same method as for the numerical *p*/*d* mode separation. At each frequency, the sampling of *E* at different moments in time within a single period does not introduce any noticeable difference to this process, i.e., no change was recorded in the *C_d_* ratio (a coefficient denoting the weighting ratio of *d_xy_*—see Figure S3, Supporting Information).

To obtain the transverse profile of the normalized value of |*u_z_* | (see Figure S4, Supporting Information), the out‐of‐plane displacement was measured at 5 mm intervals from −110 to 115 mm along the *y*‐axis, at a distance 3*a* away on the right of the wave source. The normalized value of |*u_z_* | along the *y*‐axis within the frequency range 65–80 kHz was then measured. To obtain the data for elastic spin in Figure 2 (and Figure S6, Supporting Information) the displacement polarization was subsequently measured at L1‐3 and R1‐3 in succession, that is, each time leaving only a single aluminum pillar on the plate for purposes of recording the displacements perpendicular to the two neighboring sides of this pillar. The amplitude and phase information for *u_x_* and *u_y_* was then extracted at given frequency, which yields the polarization of the displacement vector (*u_x_*, *u_y_*) at a given frequency.

In‐plane dual‐phase spin sources were constructed, yielding an acoustic polarization perpendicular to aluminum plate. These broadband subwavelength chiral sources allow unidirectional elastic wave propagation on the straight OI‐TI interface and in the waveguide‐resonator structure. All experiments were performed at room temperature at least 24 h after source installation.

During experiment, *u_z_* is recorded at a set of points along OI‐TI interface from *x* = −250 to 250 mm at 5 mm intervals. This allows the collection of temporally‐resolved signals for both right‐ and left‐directed waves (see Figure S7, Supporting Information), and enables subsequent execution of 2D‐FTs of the relevant *u_z_* component on the OI‐TI interface. The average amplitude of *u_z_* and other averaged components (see Figure 3b–e) could also be obtained.

In the case of the waveguide‐resonator structure, the displacement was measured at 5 mm intervals along the straight OI‐TI interface, as well as the quantity $\ln(\bar{|u_{R}|}/\bar{|u_{L}|})$ by the same method as before (Figure 3d,e). The displacement *u_z_* is monitored over a selected rectangular region (350 × 250 mm^2^), that includes the ring resonator and the straight OI‐TI interface, at 12.5 mm (*a*/2) intervals along the *x*‐axis or at 21.7 mm (0.87*a*) intervals along the *y*‐axis. After that, both the time‐ and amplitude‐resolved mapping of *u_z_* is carried out at specific frequencies, as shown in Figure 4d,e (and Figures S9 and S10, Supporting Information).

## Conflict of Interest

The authors declare no conflict of interest.

## Supporting information

Supporting Information

Supplemental Video 1

Supplemental Video 2

## Data Availability

The data that support the findings of this study are available from the corresponding author upon reasonable request.
