# Supplementary material for: Parity‐Frequency‐Space Elastic Spin Control of Wave Routing in Topological Phononic Circuits
Source: Adv Sci (Weinh). 2024 Jul 31;11(36):2404839. doi: 10.1002/advs.202404839 (PMC11423203; doi:10.1002/advs.202404839)
Supplement: Supplementary file 1 — Supporting Information [file ADVS-11-2404839-s001.docx]

# Supporting Information

***Parity-Frequency-Space Elastic Spin Control of Wave Routing in Topological Phononic Circuits***

Yao Huang^1^, Chenwen Yang^2^, Weitao Yuan^3^, Yuxuan Zhang^1^, Yongdong Pan^1^, Fan Yang^1^, Zheng Zhong^4^, Jinfeng Zhao^1^*, Oliver B. Wright^5,6^ and Jie Ren^2^*

1 School of Aerospace Engineering and Applied Mechanics, Tongji University, 100 Zhangwu Road, Shanghai 200092, People’s Republic of China

2 Center for Phononics and Thermal Energy Science, China-EU Joint Lab on Nanophononics, Shanghai Key Laboratory of Special Artificial Microstructure Materials and Technology, School of Physics Science and Engineering, Tongji University, Shanghai 200092, People’s Republic of China

3 Applied Mechanics and Structure Safety Key Laboratory of Sichuan Province, School of Mechanics and Aerospace Engineering, Southwest Jiaotong University, Chengdu, Sichuan 610031, People’s Republic of China

4 School of Science, Harbin Institute of Technology, Shenzhen 518055, China

5 Graduate School of Engineering, Osaka University, Yamadaoka 2-1, Suita 565-0871, Osaka, Japan

6 Hokkaido University, Sapporo 060-0808, Hokkaido, Japan

E-mail: Jinfeng Zhao: [jinfeng.zhao@tongji.edu.cn](mailto:jinfeng.zhao@tongji.edu.cn);

Jie Ren: [Xonics@tongji.edu.cn](mailto:Xonics@tongji.edu.cn)

## Supplementary Note 1. Topological phase transition of the phononic crystal plate

It is well known that phononic crystals (PhCs) with *p*/*d* symmetry inversion properties exhibit a fourfold degeneracy in their band structure. Here, we design a PhC plate with an array of clover-leaf holes, as shown in Figure **S1a**. This PhC plate can attain accidental fourfold degeneracy by simply rotating the clover-leaf holes with respect to the unit-cell axes by an appropriate angle *θ*. As a result, the PhC plate symmetry can be changed from C_6_*_v_* (*θ* = 0, 60^o^) to quasi C_6_ (*θ* = 26^o^).


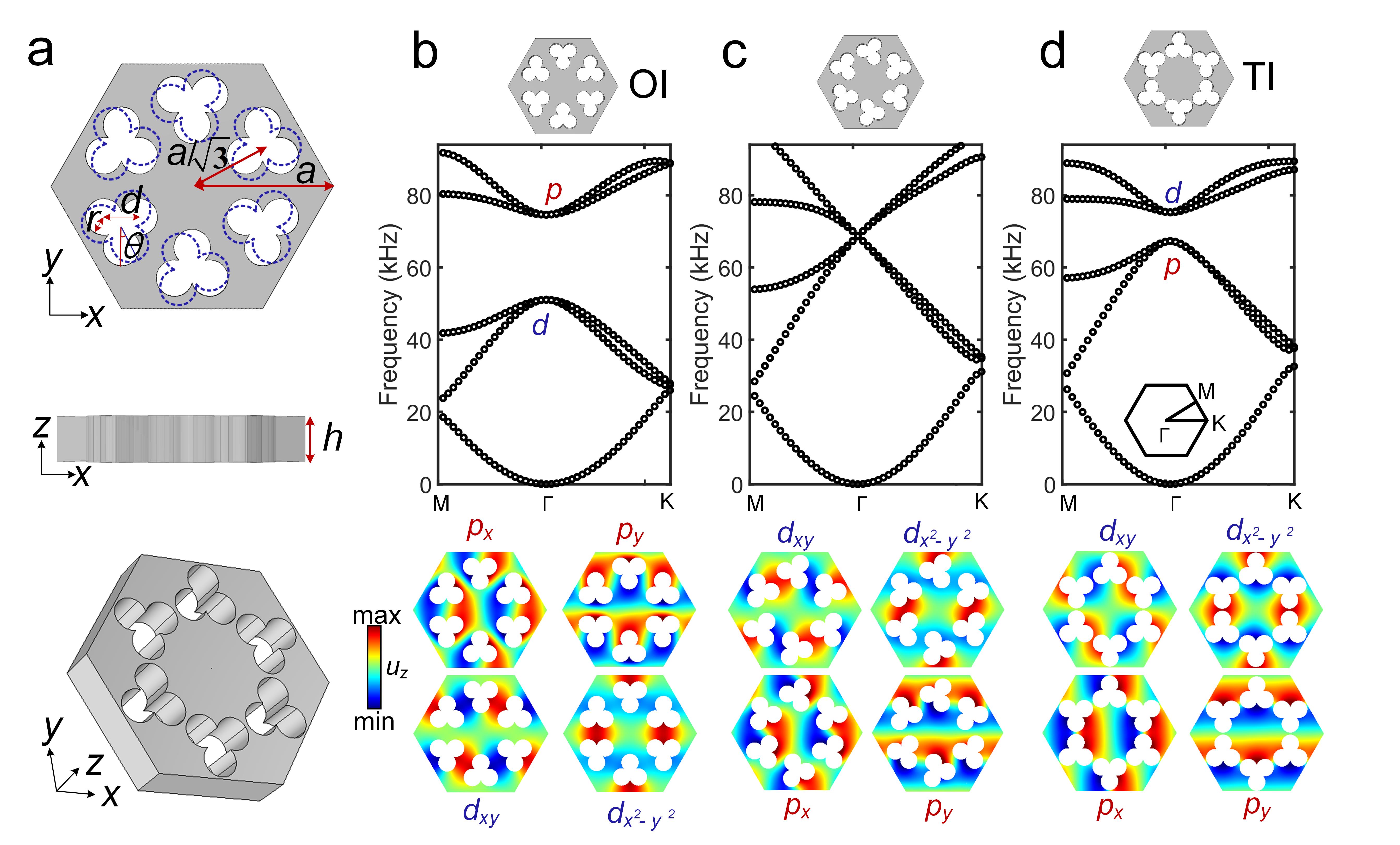


**Figure S1.** **|** **Geometry of the unit cell and the topological phase transition for the PhC plate. a** Unit cell of the PhC plate. Geometrical parameters include lattice constant *a* = 25 mm and thickness of plate *h* = 9.2 mm. The clover-leaf hole is formed from three intersecting circular holes of radius *r* = 3.25 mm, with nearest-neighbor center distance *d* = 6.24 mm. The distance from each clover-leaf hole to the center of the hexagonal unit cell is $a/\surd3$. The topological phases are controlled by changing the rotation angles *θ* of the clover-leaf holes in the unit cell. The values of the geometrical parameters *a*, *d*, *r*, *θ* and *h* are derived from the actual values measured on the samples used in experiment. **b**-**d** Simulated band structures and eigenstates of the unit cell for **b** *θ* = 0°, **c** 26° and **d** 60°. The PhC plate is an ordinary insulator (OI) when *θ* = 0°, but a topological insulator (TI) when *θ* = 60°. An antisymmetric boundary condition has been adopted for the middle plane of the PhC unit cell, so symmetrical modes are not included in the dispersion curves.

The band structures for the propagation of antisymmetric plate waves on the above PhC structure corresponding to rotation angles *θ* = 0°, *θ* = 26° and *θ* = 60°, respectively, are shown in Figures **S1b**-**d**. One can discern that the degenerate dipolar *p_x_* (*p_y_*) and quadrupolar modes $d_{x^{2}-y^{2}}$ (*d_xy_*) appear above and below the band gap, respectively, in Figure **S1b**. When *θ* = 26°, an accidental fourfold degenerate Dirac cone appears, as shown in Figure **S1c**. This fourfold degenerate Dirac point opens up when *θ* = 60°, as shown in Figure **S1d**. The *p_x_* (*p_y_*) and $d_{x^{2}-y^{2}}$ (*d_xy_*) symmetry-like orbitals are inversed on the lower and upper sides of the bandgap, respectively. This band inversion enables a topological phase transition from the trivial (ordinary insulator, OI) to the nontrivial (topological insulator, TI) case as *θ* ranges from 0° to 60°. The topological properties of our PhC plates can be characterized by topological invariants (Chern numbers *C_s_*)^1,2^. In the case of *θ* = 0 (Figure **S1b**), the *p*-symmetry bands are above the *d*-symmetry bands, and the Chern number is *C_s_* = 0, indicating that the band gap has a trivial topology. In contrast, in the case of *θ* = 60° (Figure **S1d**), the *d*-symmetry bands are above the *p*-symmetry bands, and the Chern number is *C_s_* = ±1^1,2^, leading to a magnification of the nontrivial band gap. By this change, the topological phase is transformed from an ordinary insulator (OI) with *C_s_* = 0 to a topological insulator (TI) with *C_s_* = ±1. In addition, the OI and TI exhibit relatively large overlapping band gaps, which is an advantage for experimental characterization of the TES (topological edge states) and TWGM (topological waveguide modes) in this work.

## Supplementary Note 2. Separation of the *p_y_* and *d_xy_* modes in the TES


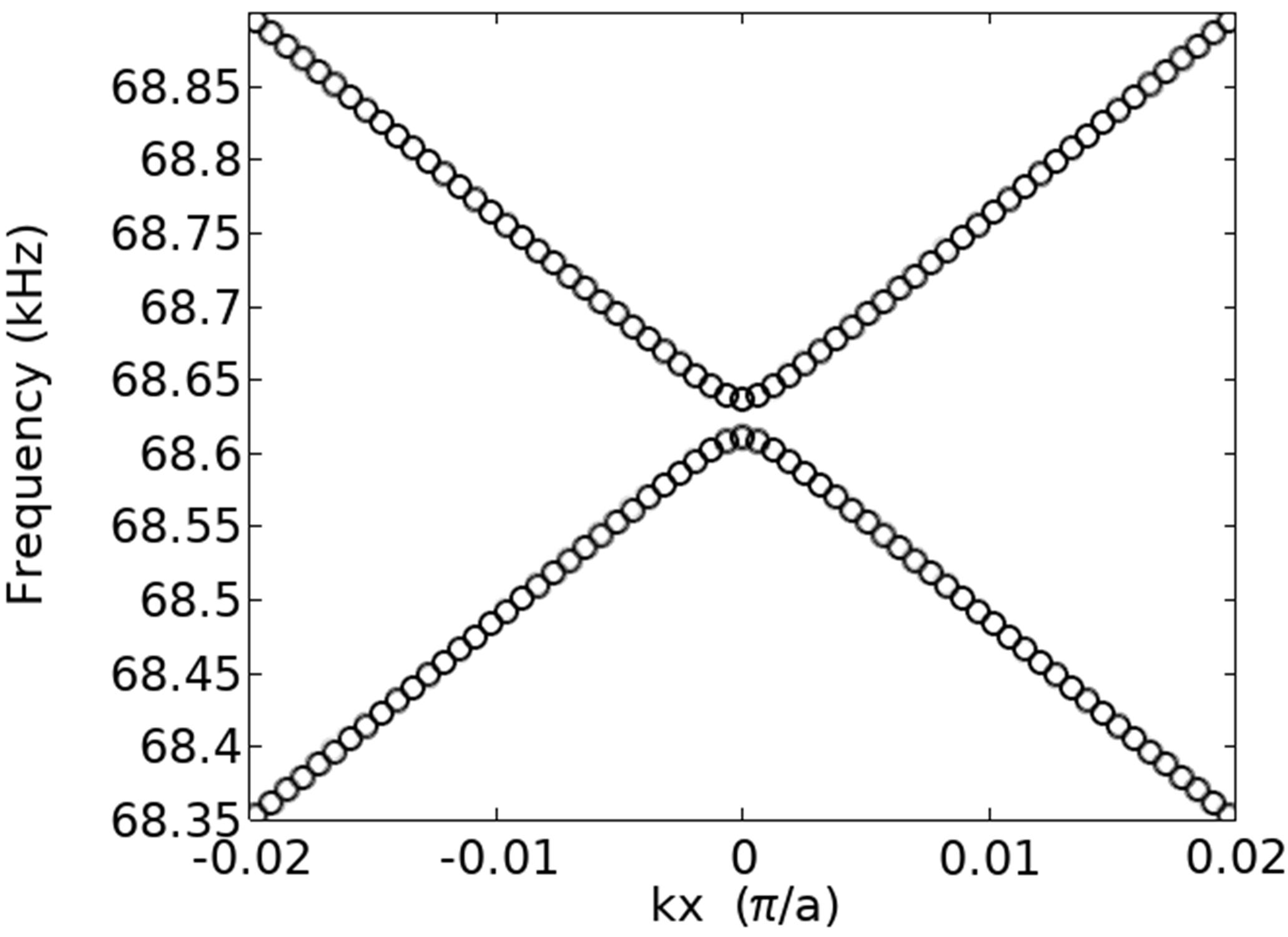


**Figure S2** **|** **A tiny gap in the frequency range of the TES dispersion.**

The zoom-in of the TES dispersion relation of Figure **S2** reveals a tiny gap from 68.611 to 68.636 kHz. The gap width of 0.025 kHz is so narrow that it has a negligible effect on the unidirectional routing of TES. The gap frequency is far removed from that of the TWGMs, so that it also has negligible impact on the unidirectional routing of TWGMs at 73.4 and 74.6 kHz.

**
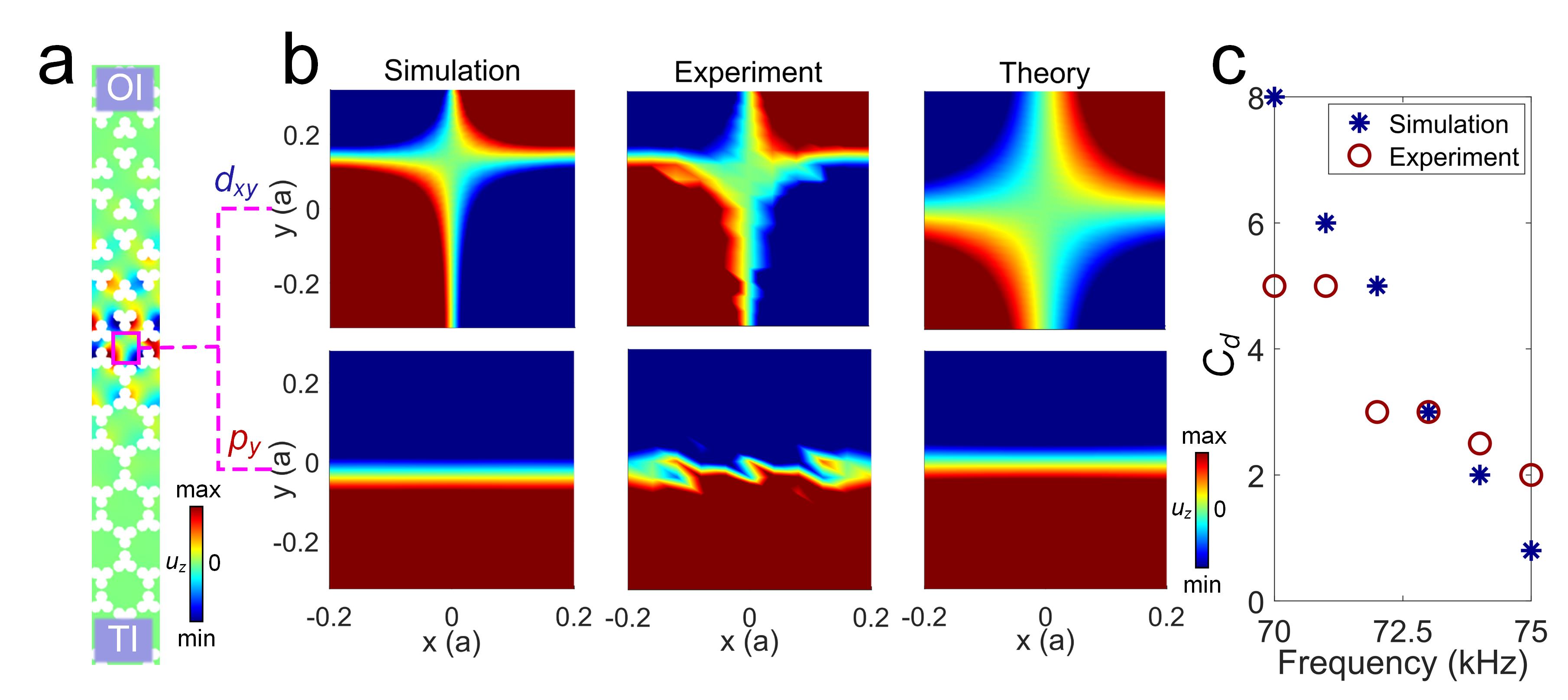
**

**Figure S3 |** **Decomposing the TES into *p_y_* and *d_xy_* modes. a** Normalized color plot of the distribution of the displacement component *u_z_* at 72 kHz for the TES obtained by simulation. The displacement *u_z_* is transversely antisymmetric about the central line of the OI-TI interface. **b** Normalized color plots of *u_z_* corresponding to modes *p_y_* and *d_xy_* separated from the displacement field (purple rectangle) by simulation (left panel), experiment (middle panel), and theory (right panel). **c** Frequency dependence of the weighting coefficient of *p_y_* and *d_xy_* modes obtained by simulation (snowflake markers) and experiment (circles).

It is well known from theory that *p_y_* and *d_xy_* modes give rise to transversely antisymmetric TESs^1^. However, the experimental selective detection of *p-* and *d-*modes has seldom been reported. This has hindered the elucidation of the theoretical origin of spin AM (angular momentum). Here, we decompose the TESs into *p_y_* and *d_xy_* modes. We present the simulated displacement field of *u_z_* on a rectangular portion of the OI-TI (ordinary-insulator/topological-insulator) interface, indicated by a purple solid rectangle in Figure **S3a**. Specifically, it represents the data for *E*, where the matrix *E* is a snapshot of the displacement field *u_z_* at a given frequency. *E* can also be extracted in experiment over the equivalent region. We sample at 1 mm intervals along both the *x-* and *y*-axes, over the area defined by -0.2*a* < *x* < 0.2*a* and -0.3*a* < *y* < 0.3*a*, where *a* is the lattice constant.

The separation of the *p_y_* and *d_xy_* modes for simulations or experiment is done by a matrix transformation (left and middle panels in Figure **S3b**)^1,2^. Namely, the *d_xy_* and *p_y_* modes are individually obtained from *E*-*ET_y_*(*β*) and *G*-*GT_x_*(*β*), where *T_x_*(*β*) and *T_y_*(*β*) are rotation matrices (*β=*180°) for the *x*- and *y*-axes respectively. Specifically, *ET_x_*(*β*) flips matrix *E* up and down along the (symmetry) *x*-axis, whereas *ET_y_*(*β*) flips matrix *E* left and right along the (symmetry) *y*-axis. Now let us define a new matrix *G* = *ET_x_*(*β*)-*ET_y_*(*β*). Matrices for *d_xy_* and *p_y_* modes can then be obtained from *E*-*ET_y_*(*β*) and *G*-*GT_x_*(*β*), respectively, as shown in Figure **S3b**.

We measured the time-resolved profile of *u_z_* at 1 mm intervals along both the *x*- and *y*-axes within the same area as in the simulation. The matrix *E* at a given frequency can be retrieved, which enables the separation of the *p_y_* and *d_xy_* modes through matrix transformation (middle panel of Figure **S3b**). Evidently, the experimental and simulated results agree well. The equivalent theoretical result is shown in the right-hand panel of Figure **S3b**, giving fair agreement. The horizontal symmetry axis of the *d_xy_* modes is a little shifted in the theory in comparison to the other results. This is in accord with the slight “spin up/down/up” (*k_x_*>0) shifting noted in the right-hand panel of Fig. **2e** in the main text.

To extract further information on the *p_y_* and *d_xy_* modes in the TES, we multiply the *d_xy_* component obtained from matrix *E* by a coefficient *C_d_* denoting the weighting ratio for *d_xy_*. The original displacement field of the TES can be reproduced to a good accuracy for both simulation and experiment by use of a linear combination of the matrix for the *p_y_* mode and that for the *d_xy_* mode, expressed as *p_y_* + *C_d_ d_xy_*. Figure **S3c** displays the frequency dependence of the weighting ratio *C_d_* obtained by simulation (snowflake markers) and experiment (circles). The displacement field of the TES is made up of a linear combination of *p_y_* and *d_xy_* modes. *C_d_* decreases gradually as the frequency increases.

## Supplementary Note 3. Transverse decay of the displacement *u_z_* of the TES

The profile of the normalized displacement |*u_z_*| along the *y*-axis was measured to verify the frequency range of the TES and to obtain the transverse decay function *R*(*y*). A single PZT-5H disk (radius *r* = 2 mm and thickness *e* = 0.5 mm) is attached to the TI-OI interface. A 5-cycle toneburst signal centered at 72 kHz is applied to it for excitation of asymmetric plate waves. A laser Doppler vibrometer is used to measure *u_z_* along the *y* axis at a distance 3*a* away from the wave source. In this way, we can obtain a time-resolved profile of *u_z_* along a vertical line, and then retrieve the amplitude of *u_z_* at a given frequency.


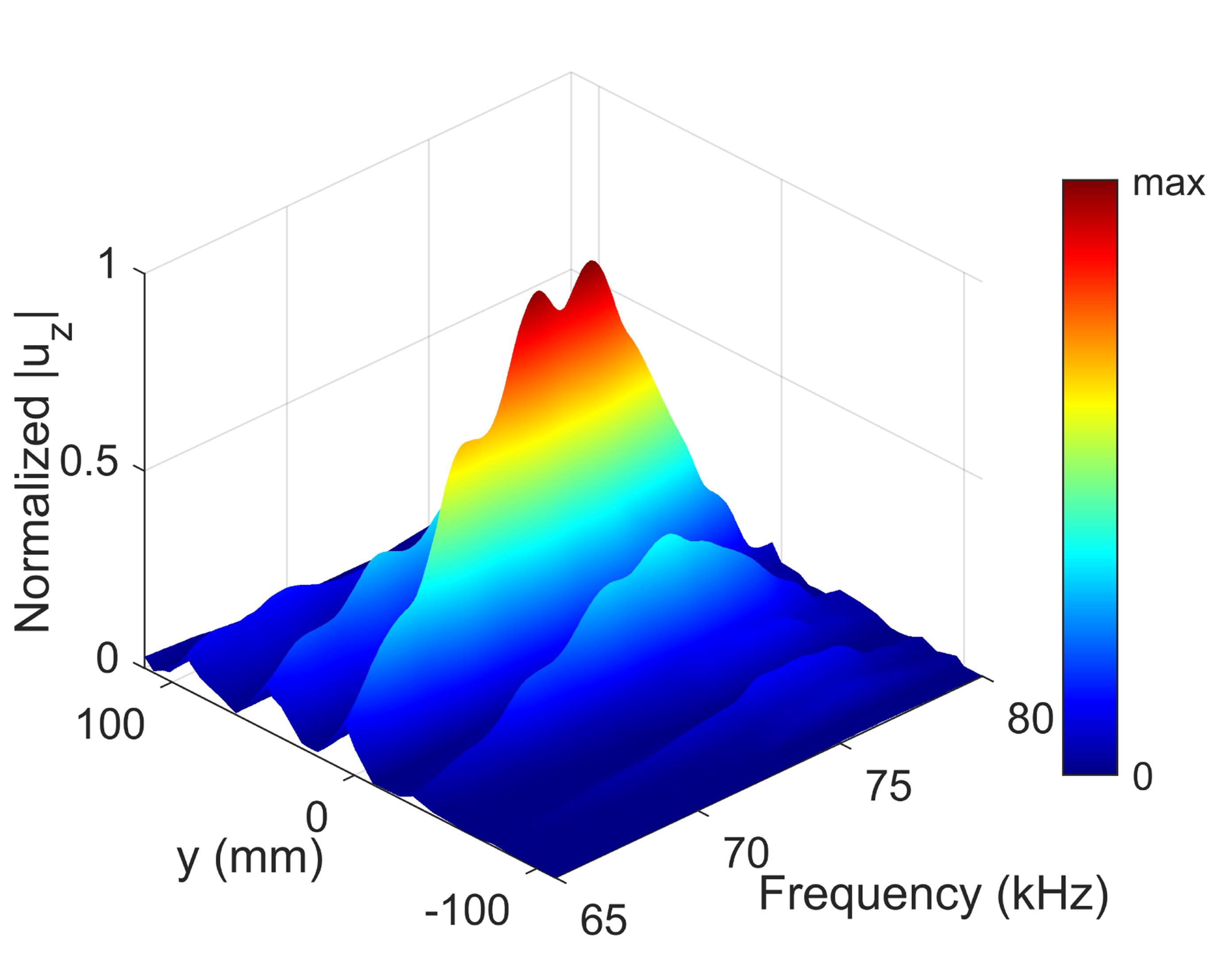


**Figure S4 |** **Measured profile of normalized |*u_z_*| along the *y*-axis of the linear topological interface.**

We show the normalized profile of |*u_z_*| vs both frequency and distance along the *y* axis in Figure **S4**. It can be seen that the elastic waves are highly localized around the OI-TI interface (*y* = 0) over the broadband frequency range of the TES (70-75 kHz). In contrast, the normalized |*u_z_*| decays relatively slowly with distance outside this frequency range. At 72 kHz, the measured spatial decay of |*u_z_*| can be fitted to the form *R*(*y*) = exp(-0.62|*y|*/*a*). Utilizing this experimental function *R*(*y*), we assume that the out-of-plane displacement field of the TES can be written in the form $u_{z}=\left[ \zeta e^{i\varphi}u_{p}(x,y)+u_{d}(x,y) \right]R(y)$ for the theoretical derivation of elastic spin *S_z_* in Supplementary Note **4**, where *u_p_* and *u_d_* are the displacement fields of the *p_y_* and *d_xy_* modes, as shown in Eq. (8). The phase factor *e^iφ^* accounts for a phase difference between the *p_y_* and *d_xy_* modes.

## Supplementary Note 4. Elastic spin density in a PhC with *p*/*d* symmetry inversion

**Effective Hamiltonian for a PhC with *p*/*d* symmetry inversion**

For application to our PhC plate, elastic waves in such an inhomogeneous medium are governed by $\left(\lambda+\mu\right)\nabla\left(\nabla\cdot u\right)+\mu\nabla^{2}u+\nabla\lambda\left(\nabla\cdot u\right)+\nabla\mu\times[\nabla\times u]+2\left(\nabla\mu\cdot\nabla\right)u+\rho\omega^{2}u=0$^4^, with displacement vector ***u*** = ***u***(***r***), Lame’s constants *λ*=*λ*(***r***), *μ*=*μ*(***r***) and mass density *ρ*(***r***). These quantities all exhibit a periodic distribution in coordinate ***r***. According to Bloch’s theory, the displacement distribution for single-frequency elastic waves in a PhC plate obeys ***u***(***r***)=***u*_0_**e*^i^*(***^k^*·*^r^***^-^*^ωt^*), where **k** is the wave vector and *ω* is the angular frequency. Following ***k***·***p*** perturbation theory, the elastic wave equation can be transformed into the following eigenvalue equation^5^:

$H_{eff}|\psi\rangle=\omega'|\psi\rangle$, (1)

where *H*_eff_ is an effective Hamiltonian, $\left| \psi\right\rangle$ is the eigenvector, and *ω'* stands for the angular frequency related to the initial eigenfrequency *ω*_0_.

With this new basis (*p*_+_, *d*_+_, *p*-, *d*-)*^T^*, where *p*_±_ = *p_x_*±*ip_y_* and *d*_±_ = *d_x2-y2_*±*id_xy_*, many authors have demonstrated that the effective Hamiltonian for a PhC exhibiting *p*/*d* symmetry inversion^1,6-8^ can be written as

$H_{eff}=\left(\begin{matrix} M+Bk^{2} & Ak_{+} & 0 & 0 \\ A^{*}k_{-} & -M-Bk^{2} & 0 & 0 \\ 0 & 0 & M+Bk^{2} & Ak_{-} \\ 0 & 0 & A^{*}k_{+} & -M-Bk^{2} \end{matrix}\right)$ , (2)

where *k*_±_ = *k_x_*±*ik_y_*, ignoring higher-order terms in *k_x_* and *k_y_* such as *k_x_*^2^- *k_y_*^2^ or *k_x_k_y_* in the off-diagonal blocks. *M* = (*f_p_*-*f_d_*)/2 is the frequency difference between modes *p_x_*/*p_y_* and *d_x_*_2−_*_y_*_2_/*d_xy_* at the *Γ* point in **k**-space, which takes either a positive or a negative value, as shown in Figure **S1b** or **S1d**, further illustrating the band inversion. The value of *A* is pure imaginary, and *B* is usually negative. Each eigenvector corresponding to Eq. (2) can be expressed in the form

, (3)

with eigenstates .

By analysis of Eq. (2), one can obtain two non-normalized eigenvectors:

$\left| 1 \right\rangle=(\zeta e^{i\varphi_{1}},1,0,0)^{T}/\sqrt{2}$,

$\left| 2 \right\rangle=(0,0,\zeta e^{i\varphi_{2}},1)^{T}/\sqrt{2}$, (4)

where $\zeta=\left[(Bk^{2}+M)\pm\sqrt{A^{2}k^{2}+(Bk^{2}+M)^{2}}\right]/iAk$ is a real number and *A* is purely imaginary^5,6^. Equation (4) implies that the quantities  corresponding to $p_{+}$ (or corresponding to $p_{-}$) and corresponding to $d_{+}$(or corresponding to $d_{-}$) have a phase difference governed by the phase factor (or ), where , , , , and$k=\sqrt{k_{x}^{2}+k_{y}^{2}}$. Therefore, using the first eigenvector $|1\rangle$ in Eq. (4), one obtains

, (5a)

where the first part corresponds to the *symmetric* mode (*S*), and the second part corresponds to the *antisymmetric* mode (*A*), as previously reported^1^. These two modes constitute the pseudospin-up state *S* + *iA* .

Similarly, using the second eigenvector $|2\rangle$ in Eq. (4), one obtains

, (5b)

where the first part corresponds to the *symmetric* mode (*S*), and the second part corresponds to the *antisymmetric* mode (*A*). The pseudospin-down state *S* - *iA* can be formed from these two modes.

From Eq. (5), one can glean that for both the pseudospin-up and pseudospin-down states, the *antisymmetric* mode can be expressed in the form .

**Elastic Spin density in a PhC with *p*/*d* mode inversion**

As required by the previously published prescription^1^, an anti-symmetric TES can be formed by hybridization of the *p_y_* and *d_xy_* modes. Referring to the relationship between *p_y_* and *d_xy_* modes in Eq. (5), we assume that the out-of-plane displacement field of the TES in our PhC is given by

$\left(-a/2<x,y<a/2\right)$, (6)

where *ζ* is a real value depending on wave number *k*. As previously mentioned, *R*(*y*)^5^ is a function that decays away from OI-TI interface along the *y*-axis.

Using thin-plate theory, the in-plane displacements are given by

$u_{x}=-z\frac{\partial u_{z}}{\partial x}=-z[\zeta e^{i\varphi}u_{p,x}+u_{d,x}]R$, $u_{y}=-z\frac{\partial u_{z}}{\partial y}=-z\left\{ \left[ \zeta e^{i\varphi}u_{p,y}+u_{d,y} \right]R+\left[ \zeta e^{i\varphi}u_{p}+u_{d} \right]R_{,y} \right\}$ . (7)

Typically, the *u_p_* of the *p_y_* mode is odd function of *y* but an even function of *x*, whereas the *u_d_* of the *d_xy_* mode is an odd function of both *x* and *y*.

Considering the elastic spin density is governed by *S_z_* = (*ρω*/2)Im(*u_x_*^*^*u_y_*-*u_x_u_y_*^*^)^9^, one can derive the following formula:

.

The first term in the square bracket shows that both the *p*/*d* mode hybridization and *R* determine the spin distribution, whereas the second term shows that the decay rate plays a modifying role.

Based on the displacement field characteristics of *p_y_* and *d_xy_*, we use sine and cosine functions to fit to *u_p_* and *u_d_* as follows:

$u_{p}=\sin\left( \frac{\pi}{a}y \right)\left[ 1+0.25\cos\left( \frac{\pi}{a}x \right) \right]$ ,$u_{d}=\sin\left( \frac{\pi}{a}y \right)\sin\left( \frac{\pi}{a}x \right)$ . (8)

Substituting Eq. (8) into Eq. (7), the spin *S_z_* along the *z*-axis can be obtained from

, (9)

where

$F(x,y)=\frac{\pi}{a}R\left[\cos\left(\frac{\pi}{a}x\right)+0.25\right]\left\{\frac{R}{2}\frac{\pi}{a}\sin\left(\frac{2\pi}{a}y\right)+R_{,y}\sin^{2}\left(\frac{\pi}{a}y\right)\right\}$.

*R*(*y*) = exp(-0.62|*y*|/*a*) is obtained by fitting to the experimental results, as shown in Supplementary Note **3**.

Equation (9) contains a position-related term *F*(*x*, *y*), whose sign can be determined at a specified position. In this equation, the term is related to the wave number.

Notably, the sign of *S_z_* can be locked with the pseudospin-up state *S* + *iA* or the pseudospin-down state *S* - *iA* at a specific position. For example, according to Eq. (5), at a specific position one can suppose

$u_{x}=u_{Sx}+iu_{Ax},u_{y}=u_{Sy}+iu_{Ay}$, (for pseudospin-up) $u_{x}=u_{Sx}-iu_{Ax},u_{y}=u_{Sy}-iu_{Ay}$, (for pseudospin-down) (10)

where the *u_Sx_*, *u_Sy_* and *u_Ax_*, *u_Ay_* are the displacement distributions for symmetric and antisymmetric modes, respectively, and they are taken as real values for simplicity. Then for the pseudospin-up state,

$S_{z}=\frac{\rho\omega}{2}\mathrm{Im}\left(u_{x}^{*}u_{y}-u_{x}u_{y}^{*}\right)=\rho\omega(u_{Sx}u_{Ay}-u_{Sy}u_{Ax})$, (11a)

whereas for the pseudospin-down state,

. (11b)

From Eq. (11), it is obvious that the overall *S_z_* is locking to the pseudospin states. In combination to the position-related term, *S_z_* and normalized *s_z_* of the TES are shown in Fig. **2** of the main text. The “spin up/down/up” and “spin down/up/down” configurations are locking to the pseudospin-up and -down states, respectively.

This theoretical analysis is fairly complex, but the basic ideas are relatively simple: both pseudospin and intrinsic spin AM are locking to the wave direction or to the momentum of the TES, so that the pseudospin and the intrinsic-spin AM must exhibit a one-to-one relationship.


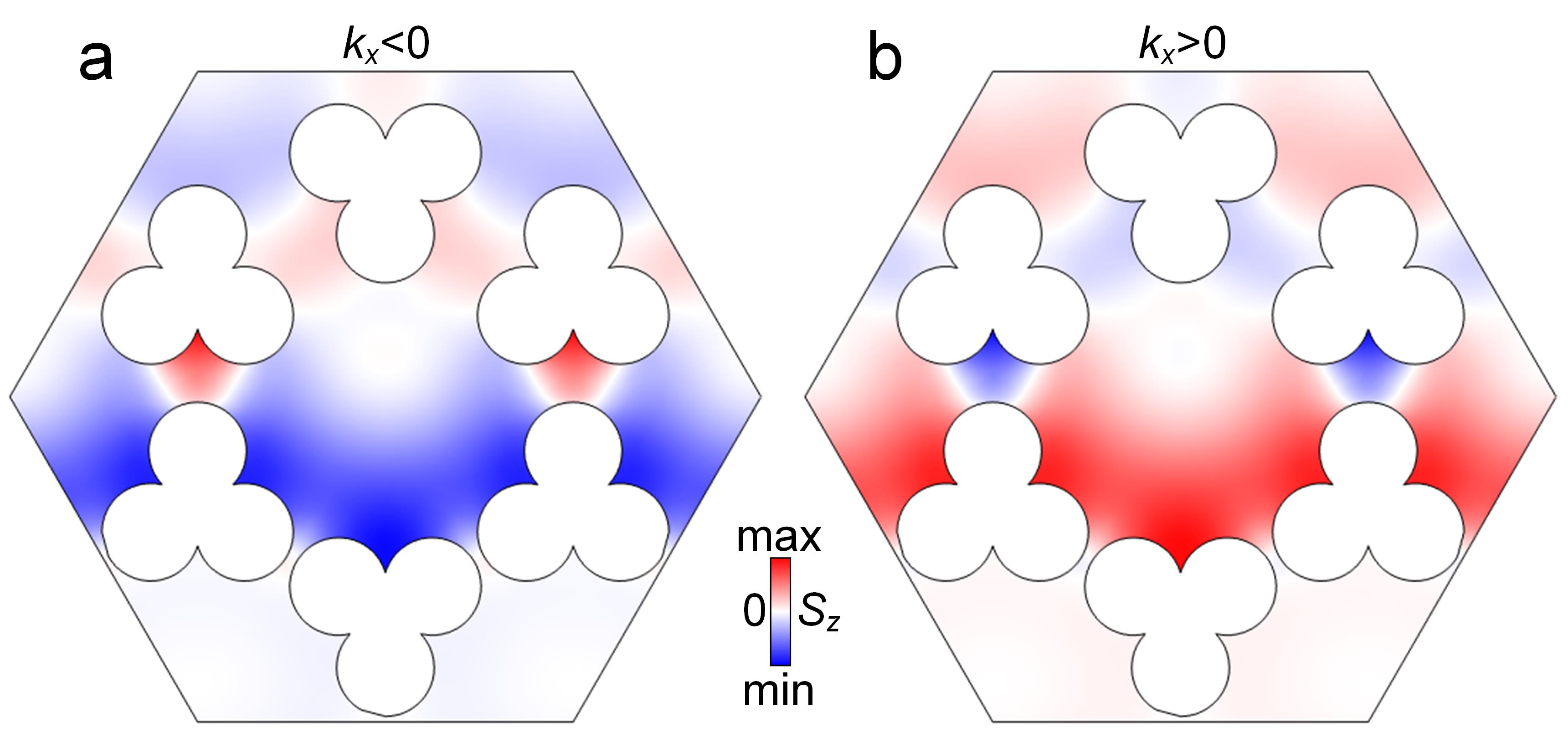


**Figure S5 |****Simulated distributions of the spin *S_z_*.** Simulated *S_z_* (i.e., not normalized) for *f* = 72 kHz at the interface (upper surface of plate) corresponding to Fig. 2d in the main text. **a** *k_x_*<0. **b** *k_x_*>0.

A significant elastic field amplitude is visible below the center of the hexagonal unit cell in Fig. **2c** of the main text, whereas the same regions in Fig. **2d** of the main text show that the normalized quantity *s_z_* approaches extrema. The in-plane energy flow evident in Fig. **2c** in the main text shows a relatively small in-plane displacement near the bottom of the hexagonal unit cell, which suggests that this polarization component dominates small *S_z_* when it takes such small values.

There is a noticeable difference between the spin *S_z_* in Figure **S5** and the normalized spin *s_z_* in Fig. **2d** of the main text. Spin *S_z_* represents the real spin angular momentum of the elastic vibration, related to the mechanical torque. The pale regions in Figure **S5** indicate that *S_z_* is relatively small in these regions, and it is hard to distinguish a macroscopic spin distribution that exhibits both positive and negative spin polarity. It is for this reason that we plot *s_z_* instead of *S_z_* in Fig. **2d** of the main text. The quantity *s_z_* not only clearly reflects the spin-up and spin-down conditions, but also the degree of in-plane elliptical polarization at different spatial positions (—circular polarization occurs when |*s_z_*|=1). At the bottom of the hexagonal unit cell in Figure **S5**, *S_z_* is relatively small, making it difficult to distinguish the sign of the spin. At the bottom of this unit cell in Fig. **2d** of the main text, one can clearly discern the sign of *s_z_*, taking values close to ±1. This does not imply that *S_z_* is close to a maxim, but rather represents the near-circular-polarization condition caused by the similar values of *u_x_* and *u_y_*.

## Supplementary Note 5. Experimental measurement of elastic spin


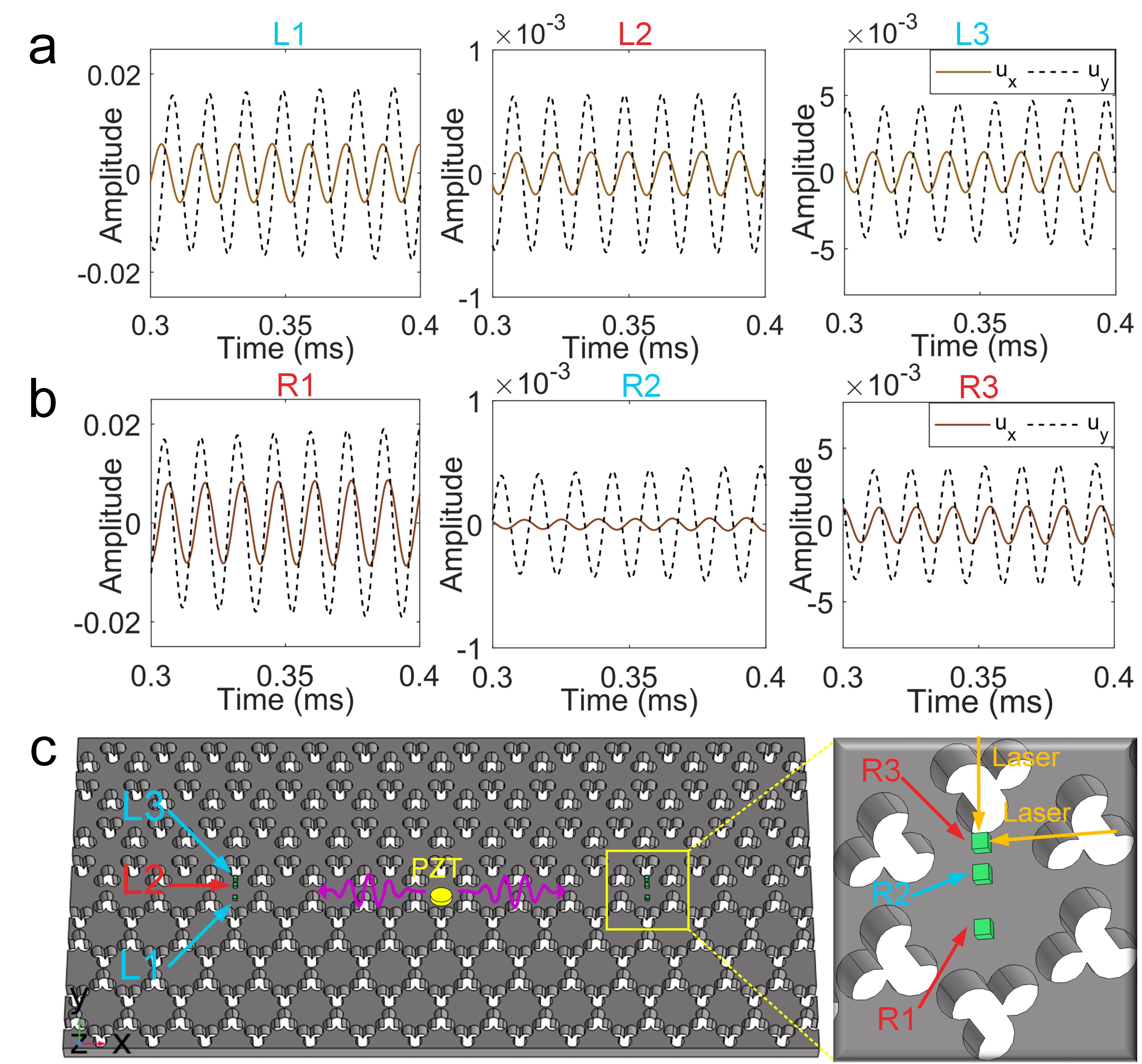


**Figure S6 | Experimental setup and results for the in-plane displacement at selected points on a linear TES. a-b** Measured profiles of *u_x_* (brown solid line) and *u_y_* (black dashed line) at L1-3 and R1-3 on the left- and right-hand sides of the wave source, respectively. **c** Experimental measurement of the elastic spin on the OI-TI interface for both left- and right-going waves at 73 kHz.

To measure the elastic spin, we again make use of the PZT-5H disk source (radius *r* = 2 mm and thickness *e* = 0.5 mm) on the OI-TI interface to generate both left- and right-going waves, as shown in Figure **S6c**. Likewise, as shown by the simulation in Fig. **2a** of the main text, we select two centerlines on the OI-TI interface on the right- and left-hand sides of the wave source, respectively. Representative points R1-3 and L1-3 are chosen, respectively. The subwavelength cube (2×2×2 mm^3^) is fixed at each point. We then measure the displacement perpendicular to two adjacent vertical planes of the cube, which allows the measurement of local in-plane displacement components *u_x_* and *u_y_*. For interference suppression, only a single cube is used at any one time on the interface to record *u_x_* and *u_y_*.

The measured profiles of *u_x_* (brown solid line) and *u_y_* (black dashed line) at positions L1-3 are given in Figure **S6a**. At L1, *u_y_* shows a ~*π*/2 phase delay compared to *u_x_*, implying a clockwise polarization of the (*u_x_*, *u_y_*) displacement pair and a negative elastic spin *S_z_*. We obtain the opposite situation at L2 (*S_z_* > 0), and a similar situation at L3 (*S_z_* < 0). This indicates the spatial dependence of elastic spin on the local environment. In Figure **S6b**, the situation at R1-3 is exactly opposite to that at L1-3, illustrating clearly the spin-momentum locking relationship. We label the sign of *S_z_* by red (+) and cyan (-) arrows in Figure **S6c**. In both experiment and simulation, the spin-momentum locking occurs over broad frequency range of the TES.

## Supplementary Note 6. Spin-based unidirectional transmission on the OI-TI interface

In Fig. **2d** in the main text, opposite spins are evident at L1-3 or R1-3 on the centerline of the hexagonal unit cell along the *y*-axis. The normalized spin |*s_z_*| in the middle and lower regions takes a value close to 1. These two regions on the OI-TI interface with opposite *s_z_* are ideally suited for installation of a circularly-polarized chiral source, in both experiment and simulation.


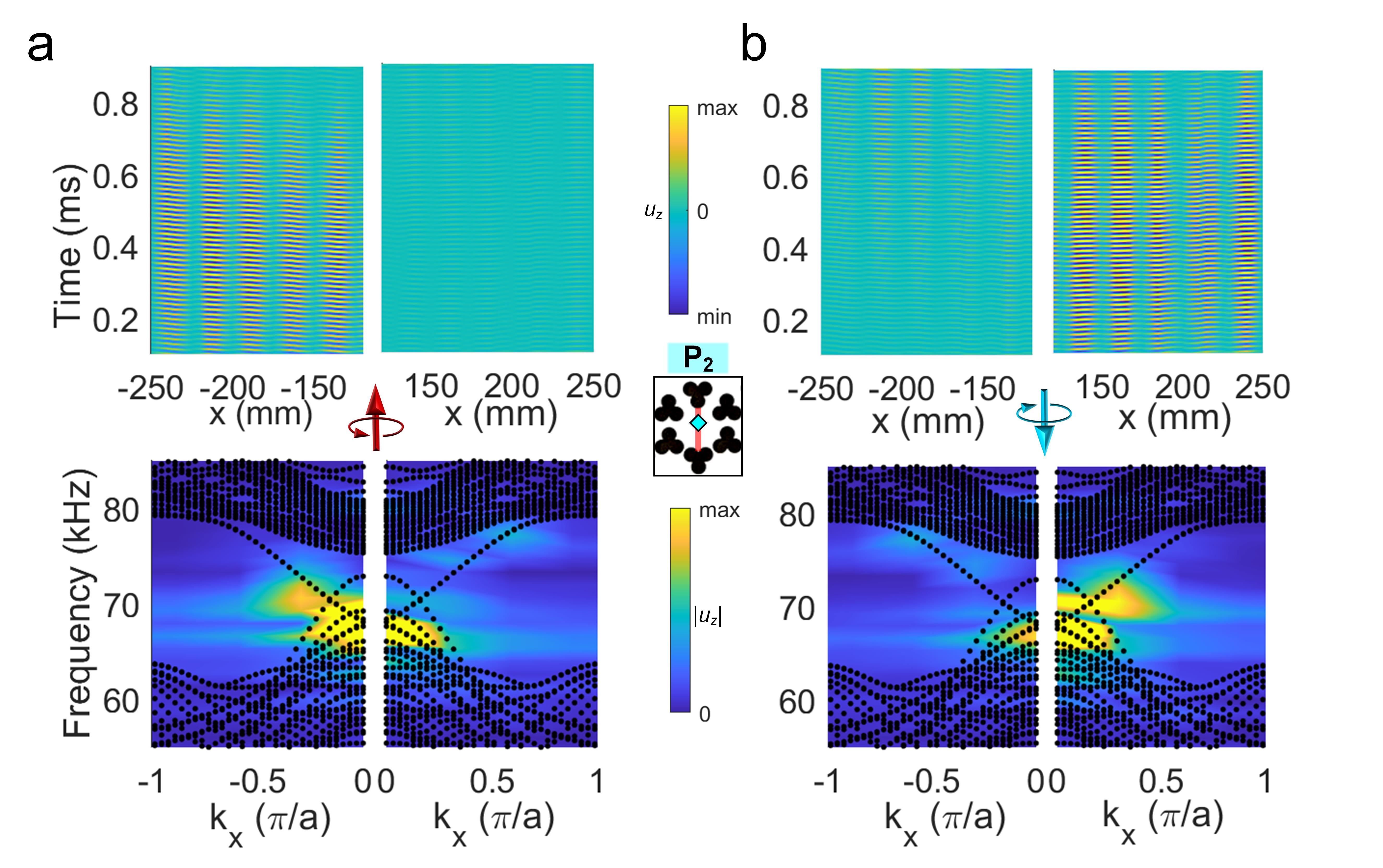


**Figure S7 | Experimental results for unidirectional routing when the chiral source is located at P_2_ on the linear OI-TI interface. a** Mapping of the measured *u_z_* vs time and along the *x*-axis (upper panel) by a spin-up source at 72 kHz. The 2D-FT spectrum of *u_z_* (lower panel). Black dots represent the simulated dispersion curves of the supercell comprising the OI-TI interface. **b** Experimental counterparts of **a** by use of a spin-down source.

In the experiment, after the chiral source installation at *x* = 0, we measure *u_z_* along the OI-TI interface from *x* = −250 to 250 mm at intervals of 5 mm. This allows us to obtain the time evolution of *u_z_* at each point. We then calculate the 2D-FT (Fourier transform) of the measured *u_z_* and extract the FT-amplitude components at different target frequencies. With the source at P_2_, Figure **S7a** (upper panel) shows a spin-up chirality *u_z_* map at 72 kHz, which indicates unambiguously a left-going wave (*k_x_* < 0). The 2D-FT helps us further confirm this unidirectional routing of the TES, as shown by the lower panel of Figure **S7a**. One can see that hot spots in the 2D-FT are widely distributed over the dispersion of the TES, and that the 2D-FT component for left-going waves is larger than for its right-going wave counterpart over the broad frequency range of the TES. Figure **S7b** presents the corresponding results when using the spin-down source at point P_2_. The results are reversed in comparison to the spin-up source, showing unidirectional routing of the TES towards the right-hand side. The spin-momentum coupling mechanism is thus demonstrated at point P_2_.

In the region between the spin source (*x* = 0) and the terminal boundary (at *x* = ±250 mm), we take the average amplitude of *u_z_* within a length of 3*a* on the right-hand (left-hand) sides of the wave source to obtain (). The experimental profiles of ln(/) are given in Figs. **3d** and **3e** in the main text for a chiral source located at P_1_ or P_2_.


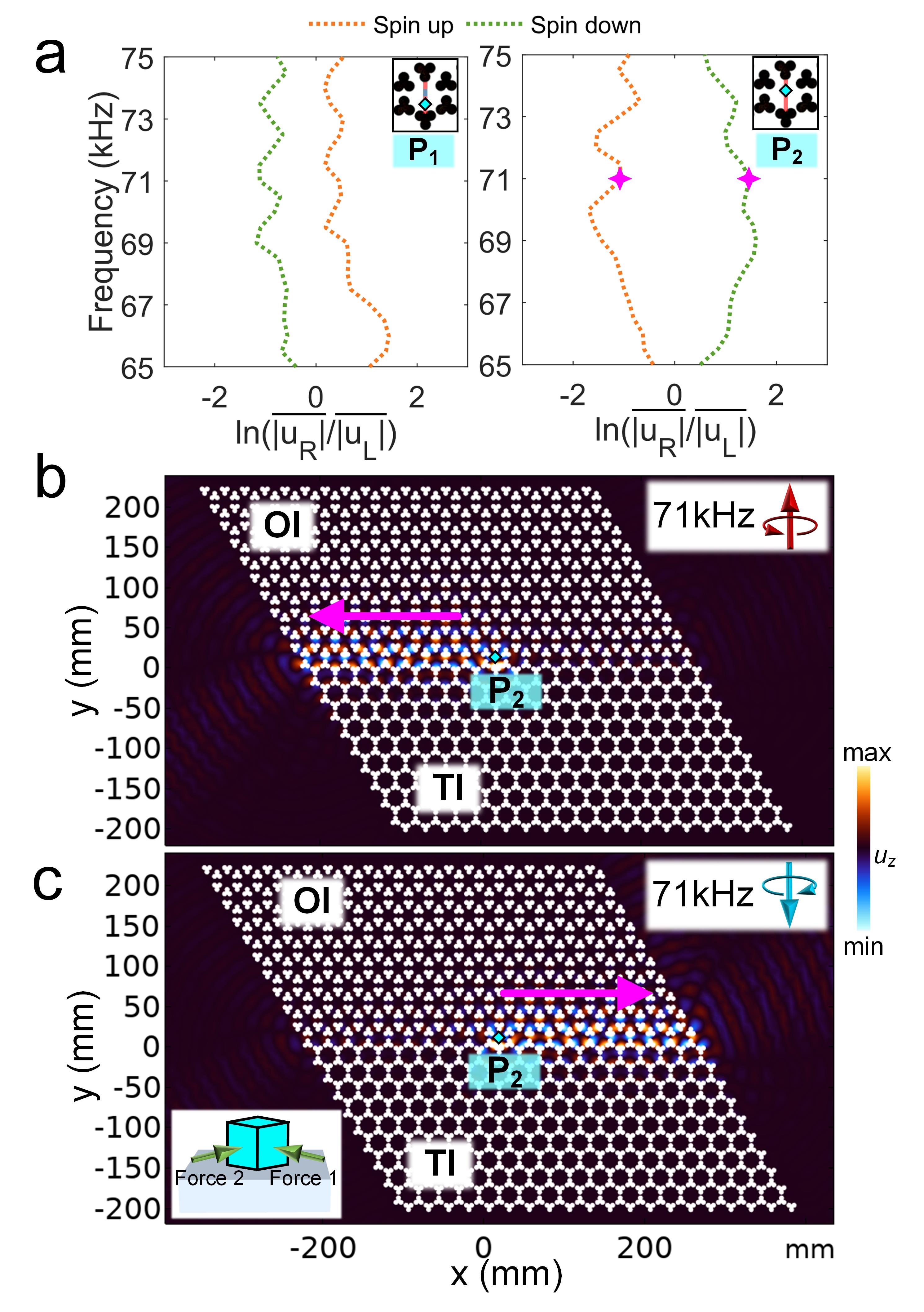


**Figure S8 | Numerical results for unidirectional routing for a chiral source located at P_1_ or P_2_ on the linear OI-TI interface. a** Simulated profiles of ln(/) for spin-up (orange line) and spin-down (green line) sources at positions P_1_ (left panel) and P_2_ (right panel), respectively. Simulated distribution of *u_z_* at 71 kHz with a spin-up source (**b**) and a spin-down source (**c**) at point P_2_.

Numerical simulations are performed using a geometry similar to that of the experimental setup. In order to save on computation cost, the simulations are conducted in the frequency domain. The simulated value of ln(/) at point P_1_ indicates a stable unidirectional transmission over a wide frequency range 65-75 kHz by use of either spin-up (orange line) or spin-down (green line) chiral sources in Figure **S8**. The simulated maximum value of ln(/) at point P_2_ reaches ~1.8 in 69-73 kHz. The simulations are consistent with the experimental results in Figs. **3d** and **3e** in the main text. Overall, both experiment and simulations are consistent with the theoretically calculated spin at points L1-2 and R1-2 in Figure **2** of the main text. The wave propagation direction can be inverted by excitation at different positions on the structure by simply altering the polarization of the spin source.

## Supplementary Note 7. Unidirectional routing in the waveguide-resonator structure


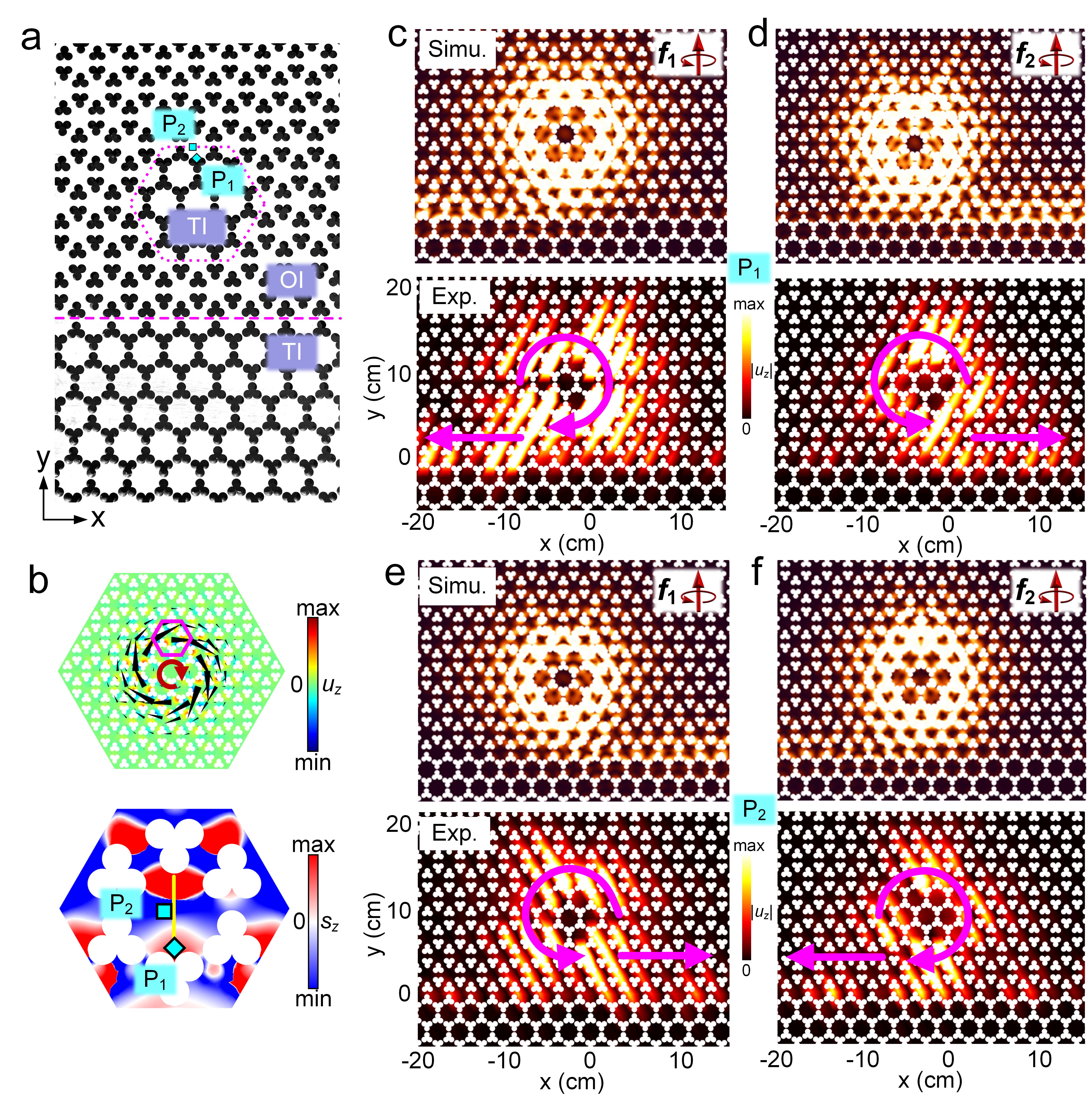


**Figure S9 |** **TWGM and waveguide-resonator unidirectional routing. a** Waveguide-resonator structure with a straight waveguide OI-TI interface (purple dashed line) coupled to the hexagonal ring resonator (purple dotted line) made of TI embedded in OI. **b** Distribution of *u_z_* for the TWGM at an eigenfrequency of *f*_2_ = 73.4 kHz (upper panel) and distribution of normalized spin *s_z_* (lower panel) in a defined region (purple solid line) at the upper edge of the ring resonator, both obtained by simulation. **c** Simulated distribution of |*u_z_*| at *f*_1_ = 74.6 kHz (upper panel), together with the experimental distribution of|*u_z_*| at *f*_1_ = 74.4 kHz (lower panel), both obtained using a spin-up source at P_1_. **d** Simulated (upper panel) and experimental (lower panel) displacement distribution of |*u_z_*|, both at *f*_2_ = 73.4 kHz, with a spin-up source at P_1_. **e** Simulated distribution of |*u_z_*| at *f*_1_ = 74.6 kHz (upper panel), together with the experimental distribution of|*u_z_*| at *f*_1_ = 74.1 kHz (lower panel), both obtained using a spin-up source at P_2_. **f** Simulated (upper panel) and experimental (lower panel) displacement distribution of|*u_z_*|, both at *f*_2_ = 73.4 kHz, obtained with a spin-up source at P_2_.

Here we construct a hexagonal ring resonator of edge length 2*a* that features two TWGMs within the bandgap of the antisymmetric plate waves. We set the distance between the ring resonator and the straight OI-TI interface to be √3a, since a significantly smaller distance would cause unwanted leakage of the wave energy towards both the right- and left-hand sides, whereas a significantly larger distance would not allow sufficient acoustic energy coupling into the straight OI-TI interface. Our choice of geometry facilitates the observation of unidirectional propagation.

Figure **S9b** shows the distribution of *u_z_* and normalized *s_z_* at *f*_2_ = 73.4 kHz. In comparison to Fig. **4b** of the main text at *f*_1_ = 74.6 kHz, the sign of *s_z_* is reversed at *f*_2_ = 73.4 kHz. If the spin-up source is situated at P_1_, the simulated results of Figure **S9c** (upper panel) show that the energy flux is directed in a clockwise direction on the ring resonator, and couples at the OI-TI interface to left-going propagation at *f*_1_ = 74.6 kHz. The experimental distribution of |*u_z_*| shown in Figure **S9c** (lower panel) fits well with the simulated result. With the same spin-up source, the simulated and experimental unidirectional routing at *f*_2_ = 73.4 kHz shown in Figure **S9d** is opposite to that of Figure **S9c**. This illustrates the frequency dependence of the spin control of TWGMs, differing from the broadband spin-momentum locking mechanism of the TESs.

To gain further understanding of the unidirectional routing for our waveguide-resonator structure, we select a second source point P_2_ (see the light-blue square in Figure **S9a**) above the first source point P_1_, chosen such that *S_z_* at P_1_ and P_2_ should be opposite to one another. In the waveguide-resonator structure, the spin-related unidirectional routing should flip when the source position is changed from point P_1_ to P_2_, as shown in Figures **S9c-d** to Figures **S9e-f**. These results are analogous those we obtained at a linear OI-TI interface.





**Figure S10 | Unidirectional routing in** **a waveguide resonator with a chiral source at point P_2_. a** Plots of frequency vs for simulation (middle panel) and experiment (right panel), where () is the average of |*u_z_*| measured along the straight OI-TI waveguide on the right-hand (left-hand) side of the TWG. Spin-up (orange line) or spin-down (green line) sources are located at point P_2_. **b** Simulated (upper panel) and experimental (lower panel) distributions of |*u_z_*| at *f*_1_ = 74.6 and 74.1 kHz, respectively. **c** Simulated (upper panel) and experimental (lower panel) distributions of |*u_z_*|, together with the in-plane energy flux vectors (purple arrows), both at *f*_2_ = 73.4 kHz. A spin-down source is used for both (**b**) and (**c**).

The relevant simulated and experimental results for the case of the spin source positioned at P_2_ are shown in Figure **S10**. As expected, the results are opposite to the case of the source positioned at P_1_. Figure **S10a** shows peaks and dips in plots of frequency vs ln(/) for both simulation (middle panel) and experiment (right panel), making use of spin-up and spin-down sources. This demonstrates the generation of TWGMs at the respective eigenfrequencies (left panel). The peak and dip frequencies in experiment are, as before, slightly shifted with respect to those in the simulations.

**
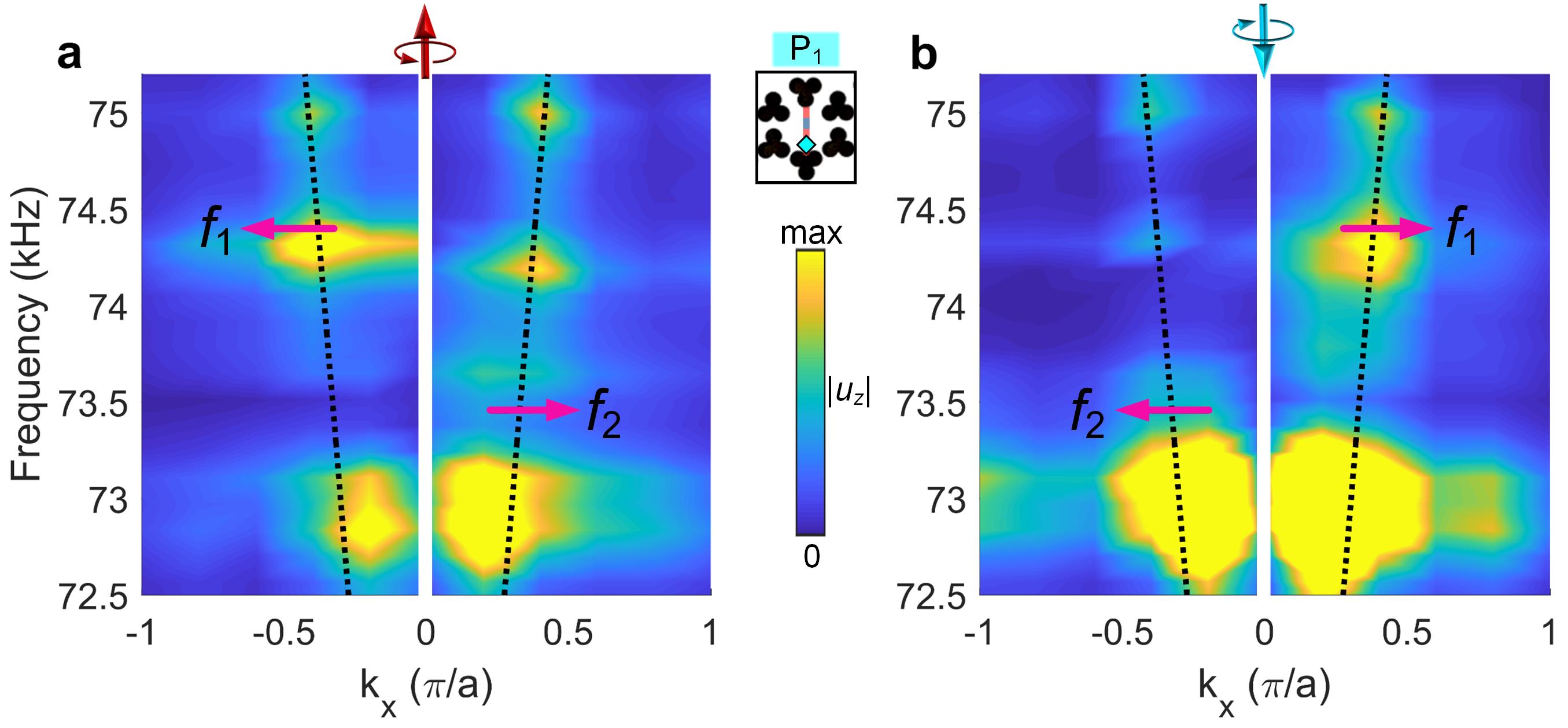
**

**Figure S11 |** **Experimental 2D-FFT spectrum of *u_z_* along the straight OI-TI waveguide with (a) a spin-up source and (b) a spin-down source, both located** **at P_1_ on the upper edge of the ring resonator.** Black dotted lines represent the calculated dispersion curves of the supercell comprising only the OI-TI waveguide.

In the experiment on the waveguide-resonator structure, we measure *u_z_* along the OI-TI interface every 5 mm over a distance of ~4*a* on the right (left) side of the ring resonator, when the chiral source is located at P_1_. Figure **S11** shows 2D-FFT spectral maps of *u_z_* for both right- and left-directed waves together with the dispersion curves of the straight OI-TI waveguide.

With the spin-up source at P_1_, as shown in Figure **S11a**, hot spots overlap the dispersion curves of the TES (dotted lines). The experimental frequencies of paired TWGMs are marked by purple arrows. Near frequency *f*_1_, the left-hand side spot is larger than its right-hand side counterpart extending from 74 to 74.7 kHz, indicating dominant left-directed wave propagation. Near *f*_2_, extending from 72.5 to 73.7 kHz, the right-hand side spot is brighter than its left-hand side counterpart, indicating dominant right-directed wave propagation. This is consistent with the orange line in the right-hand panel of Fig. **4c** in the main text.

With the spin-down source at P_1_, as shown in Figure **S11b**, the hot spot located near *f*_1_ extending from 73.7 to 74.7 kHz shows dominant right-directed wave propagation. On the other hand, near *f*_2_ the hot spot indicates dominant left-directed propagation. These results are consistent with the green profile in the right panel of Fig. **4c** in the main text, and are also consistent with the displacement distributions in Figs. **4d** and **4e** in the main text. The 2D-FFT component in Figure **S11** provides more details of the asymmetrical wave routing. This being said, the ratio is an effective indicator of the unidirectional routing.


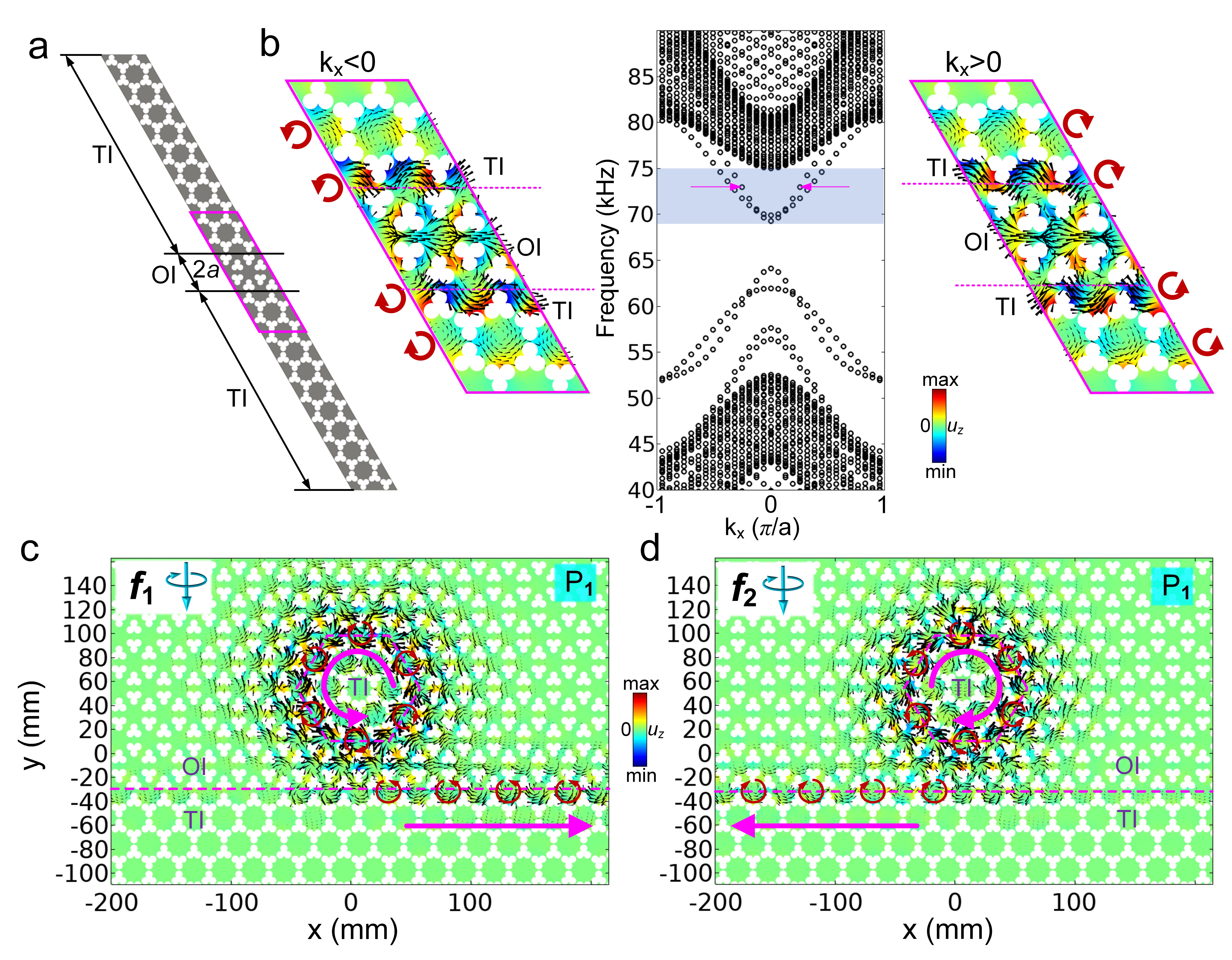


**Figure S12 |** **Sandwiched TI-OI-TI supercell and computed in-plane energy flux in the waveguide-resonator structure. a** Sandwiched TI-OI-TI supercell. **b** Computed dispersion of a TI-OI-TI supercell (middle panel), together with the distribution of *u_z_* (left and right panels) for the region denoted by the purple parallelogram in (**a)** at representative frequencies in the dispersion (purple straight arrows in the middle panel). Plots for antisymmetric plate waves correspond to the chosen supercell, and the shaded region represents a mutual band gap for the antisymmetric plate waves of the OI and TI. The pseudospin-up- and down states are associated with anticlockwise and clockwise in-plane energy flux vectors (black arrows), and are illustrated by the red circular arrows. **c-d** Distribution of *u_z_* and in-plane energy flux vector (black arrows), calculated by putting a spin-down source at point P_1_ for *f*_1_ = 74.6 kHz (**c)** and *f*_2_ = 73.4 kHz (**d)**. The purple dashed line represents the topological interface. The same notation as in **b** is used to represent the pseudospin-up and -down states and their associated in-plane energy flux vectors. The dominant wave directions along the hexagonal ring resonator and linear interface are denoted by purple circular and straight arrows, respectively.

Since the distance between lower edge of the ring resonator and the OI-TI interface is chosen to be $\sqrt{3}a$, strong coupling can occur between the respective guided modes. To reveal this coupling through simulation, another supercell is constructed as a TI-OI-TI combination, as shown in Figure **S12a**, to locally mimic the waveguide-resonator structure. The dispersion for antisymmetric plate waves for the above TI-OI-TI supercell are shown in Figure **S12b**. In contrast to the OI-TI interface shown in Fig. **2a** of the main text, there are four branches of the TES within the mutual band gap (shaded region) of the antisymmetric plate waves of the OI and TI. The distribution of *u_z_* (shown by the color scale) and that of the in-plane energy flux vector (shown by the arrows) at representative frequency along the four branches of the TES are shown in Figure **S12b**. For the two branches (*k_x_* > 0) featuring a positive slope, corresponding to right-going waves, in right panel of Figure **S12b**, one can see that the in-plane energy flux is directed in both clockwise (top TI and adjacent OI) and counterclockwise (bottom TI and adjacent OI) directions. The in-plane energy flux polarization flips at the middle of the OI. In the right panel of Figure **S12b**, these in-plane energy flux distributions reveal, in order from the top pseudospin-down state at the TI-OI interface (red circular arrow, associated with clockwise black energy-flux arrows) to the bottom pseudospin-up state at the OI-TI interface (red circular arrow, associated with counterclockwise black energy-flux arrows), that the *pseudospin-down to pseudospin-up combination* locks to the right-going wave. On the other hand, along each of the two branches (*k_x_* < 0) featuring a negative slope corresponding to left-going waves, as shown in the left panel of Figure **S12b**, the in-plane energy flux is directed in the counterclockwise (top TI and adjacent OI) and clockwise (bottom TI and adjacent OI) directions, being exactly opposite to the right-going case. In the left panel of Figure **S12b**, these in-plane energy flux distributions hence reveal, in the order from the top pseudospin-up state at the TI-OI interface (red circular arrow, associated with counterclockwise black energy-flux arrows) to the bottom pseudospin-down state at the OI-TI interface (red circular arrow, associated with clockwise black energy-flux arrows), that the *pseudospin-up to pseudospin-down combination* locks to the left-going wave.

Given this pseudospin-direction locking relationship, we now turn to the calculated *u_z_* and in-plane energy flux in the waveguide-resonator structure. Consider the experimental results in Figs. **4d** and **4e** of the main text, in which case a spin-down source is used at P_1_ at frequencies *f*_1_ and *f*_2_, respectively. Their computed *u_z_* and in-plane energy-flux vector fields are shown in Figures **S12c**-**d**, respectively. In Figure **S12c**, plotted for the frequency *f*_1_, the TI inside the ring path (purple dashed line) features a clockwise in-plane energy flux vector within the unit cell (pseudospin-down state marked by a clockwise red circular arrow), which allows for a similarly directed energy flux at the neighboring OI. The energy flux rotation direction flips on approaching the OI-TI straight interface, finally yielding a counterclockwise in-plane energy flux vector within the unit cell (pseudospin-up state marked by a counterclockwise red circular arrow) at the bottom TI below the straight OI-TI interface. One can see that, globally, the energy flows in a counterclockwise sense along the ring resonator (purple circular arrows), and couples to the right-going direction on the OI-TI straight interface. This energy flux distribution agrees well with the *pseudospin-down to pseudospin-up combination* of a right-going wave in the TI-OI-TI supercell in the right-hand panel of Figure **S12b**, i.e., the top TI-OI interface (clockwise red arrow corresponding to the pseudospin-down state) and the bottom OI-TI interface (counterclockwise red arrow corresponding to the pseudospin-up state). In Figure **S12d**, plotted at frequency *f*_2_, the in-plane energy flux vector shows exactly the opposite behavior to the case for *f*_1_. The energy flux directed from the TI, internal to the ring, to the bottom TI (below the linear interface) fits well with the *pseudospin-up to pseudospin-down combination* for left-going waves in the TI-OI-TI supercell in the left panel of Figure **S12b**, i.e., at the top TI-OI interface (counterclockwise red arrow corresponding to the pseudospin-up state) and the bottom OI-TI interface (clockwise red arrow corresponding to the pseudospin-down state). Consequently, the energy flows in a clockwise sense around the ring resonator (purple circular arrows), and couples to the left-going direction on the linear OI-TI interface. This wave propagation analysis should be of help in understanding similar optical waveguide-resonator structures based on the *p*/*d* symmetry inversion mechanism^3^.

## Supplementary Note 8. Theoretical analysis of the TWGM

The eigenmodes for paired TWGMs can be written as^5,10,11^

$\left| q,+ \right\rangle=(e^{i\varphi/2}p_{+}+e^{-i\varphi/2}d_{+})e^{iq\varphi}R(r)$,$\left| q,- \right\rangle=(e^{-i\varphi/2}p_{-}+e^{i\varphi/2}d_{-})e^{iq\varphi}R(r)$, (12)

with eigenfrequencies

$\omega_{+}=\omega_{c}+q\omega_{0}$, $\omega_{-}=\omega_{c}-q\omega_{0}$, (13)

where *ω_c_* is the center angular frequency of the band gap and *ω*_0_ is an angular frequency related to the size of ring resonator. In Eq. (12), as before, *R*(*r*) is a term representing the spatial exponential decay from the boundary of the ring resonator. The quantity *q* is the azimuthal mode number, which takes the values ±1/2, ±3/2, ±5/2, ….


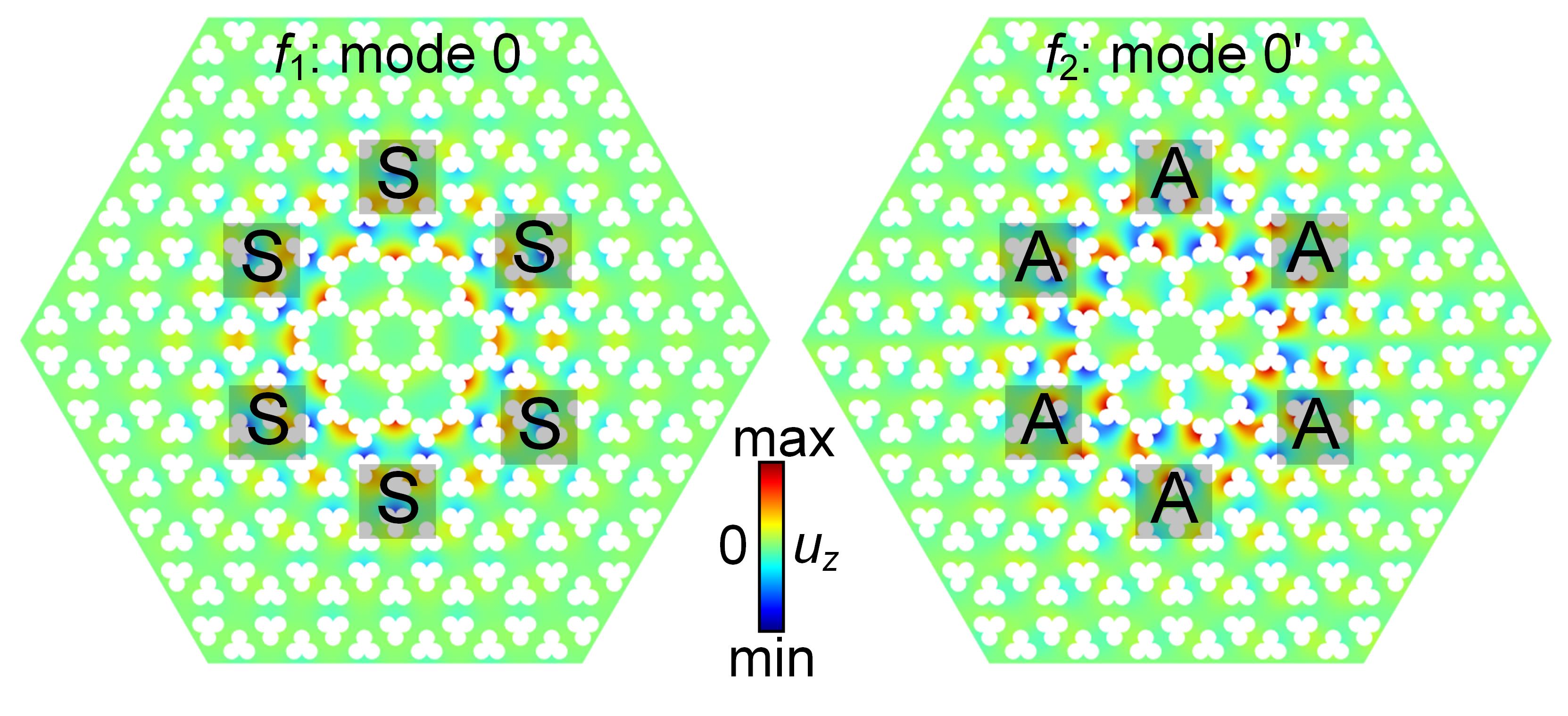


**Figure S13 |** **Simulated distributions of *u_z_* for TWGMs at frequencies *f*_1_ and *f*_2_.**

From Eq. (12), one can see that TWGMs appear in pairs due to the phononic crystal symmetry: one represents counterclockwise energy flow around the ring (state $|q,+\rangle$) at *f*_1_ whereas the other represents clockwise energy flow (state $|q,-\rangle$) at *f*_2_. However, for our hexagonal cavity resonator, the paired states are not degenerate because of the non-circular nature of our hexagonal closed loop^5^. As shown in Figure **S13**, the TWGMs in this work feature the two modes termed 0 and 0', where *q* = 1/2, *ω*_+_ = *ω*_c_ + 1/2*ω*_0_ and *ω*- = *ω*_c_ − (−1/2)*ω*_0_, the latter two corresponding to the state $|q,+\rangle$ with counterclockwise energy flow, and the state $|q,-\rangle$, with clockwise energy flow. In addition to the opposite energy flows for the paired TWGMs caused by broken spatial symmetry, time-reversal symmetry allows the TWGMs to be reversed at a single frequency, i.e., from $|q,+\rangle$ to $|q,-\rangle$ (or from $|q,-\rangle$ to $|q,+\rangle$) at *f*_1_, or from $|q,-\rangle$ to $|q,+\rangle$ (or from $|q,+\rangle$ to $|q,-\rangle$) at *f*_2_.

The excitation wave dynamics of the two TWGMs can be summarized as follows: for spin-down excitation at point P_1_, as shown in Fig. **4d** of the main text, the excited TWGM state is $|q,+\rangle$, which exhibits an anticlockwise energy flux when at frequency *f*_1_ (corresponding to *ω_+_*). At *f*_2_ (corresponding to *ω-*), the excited TWGM state is $|q,-\rangle$, which exhibits a clockwise energy flux. Accordingly, the results in Fig. **4e** of the main text show a clockwise energy flux.

Elastic spin provides another degree of freedom to tune the wave direction. When putting a spin-up source at point P_1_, the TWGM state at frequency *f*_1_ is $|q,-\rangle$ (which exhibits a clockwise energy flux at frequency *ω*-), whereas the TWGM state at *f*_2_ is $|q,+\rangle$ (with a counterclockwise energy flux at frequency *ω*_+_), which represents the opposite behavior compared to the case of a spin-down source at point P_1_. These conclusions are evident from the simulations and experimental results in Figures **S9-10**. Similarly, when the point P_2_ is selected for spin excitation, as shown in Figures **S9-10**, the situation becomes opposite to that for the case of excitation at point P_1_, owing to the opposite intrinsic spins that exist at these two positions.


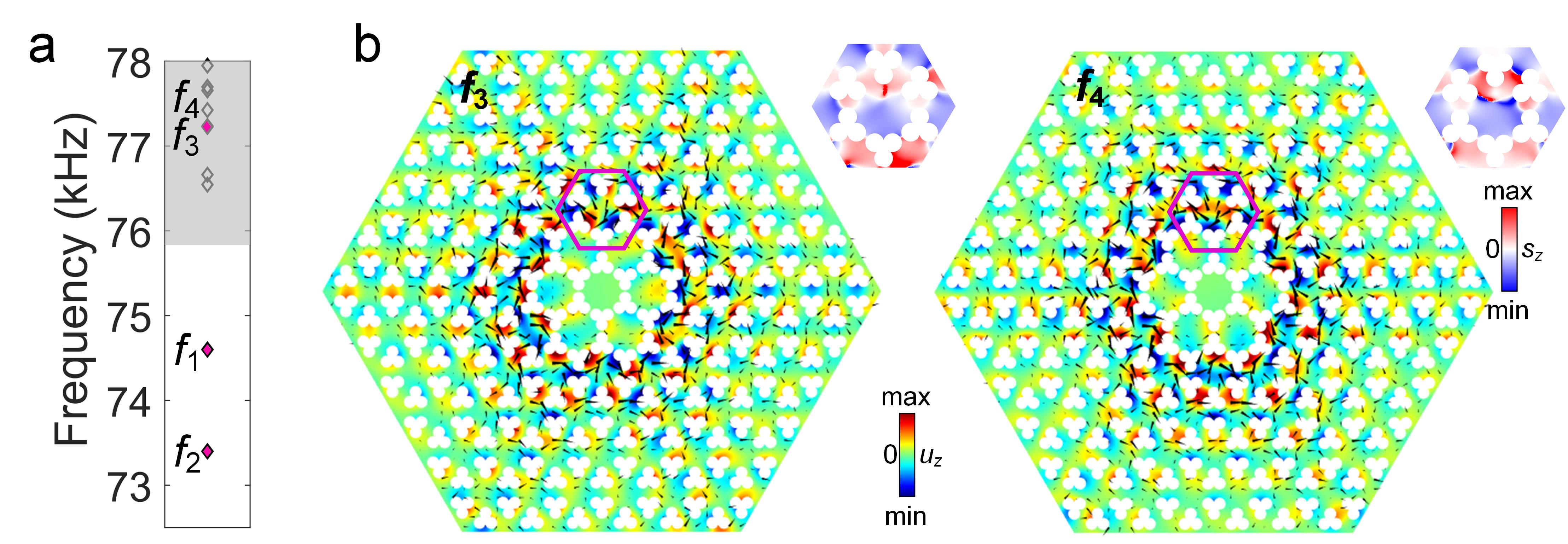


**Figure S14 |** **Simulated eigenfrequencies, distributions of *u_z_* and in-plane energy flux for the hexagonal ring resonator.** **a** Simulated eigenfrequencies. **b** Simulated distribution of *u_z_*, in-plane energy flux, and distribution of *s_z_* (inset) over a zoomed-in view of the unit cell (purple solid line) at the upper edge of the resonator, at *f*_3_ = 77.23 kHz (left panel) and *f*_4_ = 77.24 kHz (right panel).

Besides TWGMs at frequencies *f*_1_ and *f*_2_, other TWGMs can exist in the bulk band, for example at *f*_3_ and *f*_4_, as shown in Figure **S14a**. Relevant displacement distributions are shown in Figure **S14b**. The acoustic displacement is obviously localized around the hexagonal ring resonator, as is the case at frequencies *f*_1_ and *f*_2_. In addition, displacement outside the hexagonal cavity is also evident, indicating perturbations by bulk modes. The spin distribution along the centerline of the hexagonal unit cell, as shown by the upper insets, exhibits a different distribution compared to that for “spin down/up/down” or for “spin up/down/up” at *f*_1_ or *f*_2_.


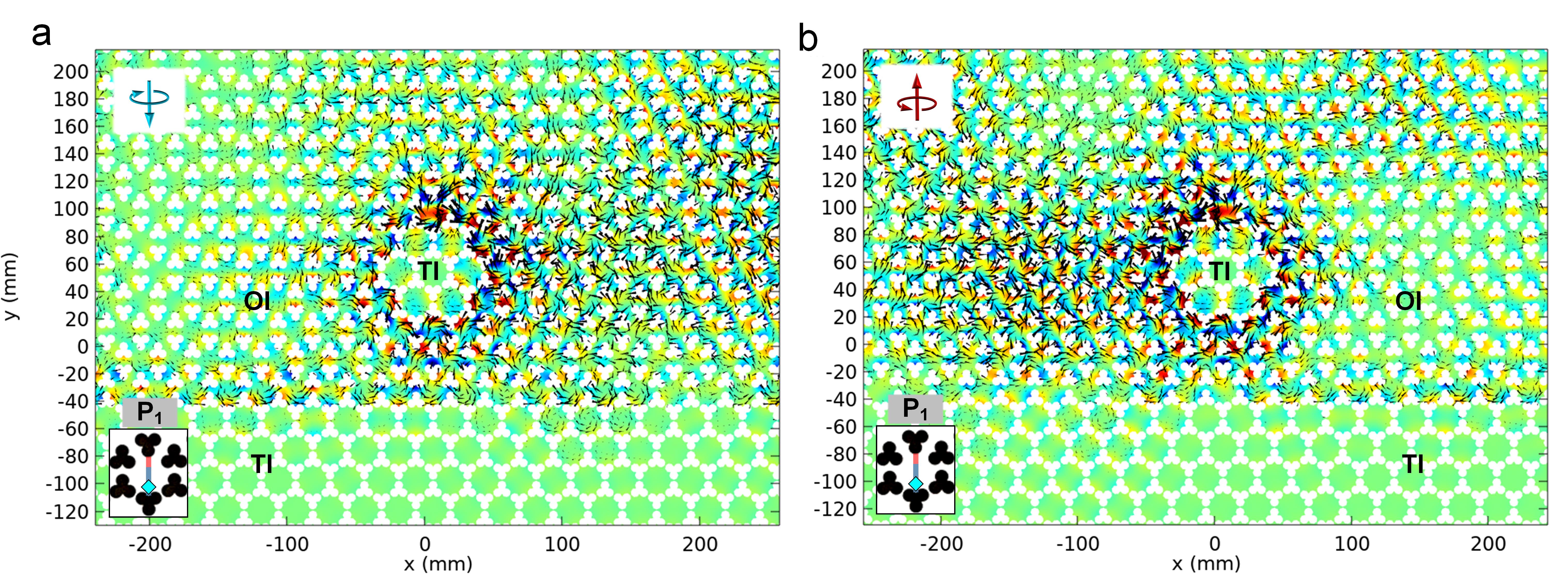


**Figure S15 |** **Simulated distribution of *u_z_* and in-plane energy flux vector.** Simulated distributions of *u_z_* and in-plane energy flux vector (black arrows) in the waveguide-resonator structure on putting (**a**) a spin-down source and (**b**) a spin-up source at point P_1_ for *f*_3_=77.23 kHz.

To clarify the wave transmission properties at frequency *f*_3_, we simulate the case for a spin-down source placed at point P_1_, as shown in Figure **S15a**. One can see that considerable energy leaks from the ring cavity into a larger portion of the OI area on both the right and left of the ring resonator. The displacement in these non-topologically protected regions indicates the influence of bulk modes at frequency *f*_3_. Asymmetric wave propagation is not observed on the straight OI-TI waveguide. When simulating a spin-up source at the same point P_1_ in Figure **S15b**, the situation becomes opposite to that of the case of using a spin-down source. However, owing to the energy leakage from the ring resonator into the OI region, asymmetric wave propagation is not expected to be efficient on the straight OI-TI waveguide. Similar simulation results are obtained by putting the spin source at P_1_ at frequency *f*_4_.

# References

1 Yu, S. Y. et al. Elastic pseudospin transport for integratable topological phononic circuits. *Nat. Commun.* **9**, 3072 (2018).

2 Yang, L. et al. Robust Fano resonance between mechanical first- and second-order topological states. *Int. J. Mech. Sci.* **236**, 107768 (2022).

3 Yang, Y. & Hang, Z. H. Topological whispering gallery modes in two-dimensional photonic crystal cavities. *Opt. Express* **26**, 21235-21241 (2018).

4 Jr, F. K. & Keller, J. Elastic wave propagation in homogeneous and inhomogeneous media. *J. Acoust. Soc. Am.* **31(6)**, 694–705 (1959).

5 Siroki, G., Huidobro, P. A. & Giannini, V. Topological photonics: From crystals to particles. *Phys. Rev. B* **96**, 041408 (2017).

6 Wu, L. H. & Hu, X. Scheme for achieving a topological photonic crystal by using dielectric material. *Phys. Rev. Lett.* **114**, 223901 (2015).

7 Li, J., Wang, J., Wu, S. & Mei, J. Pseudospins and topological edge states in elastic shear waves. *AIP Adv.* **7**, 125030 (2017).

8 Huo, S. Y., Chen, J. J., Feng, L. Y. & Huang, H. B. Pseudospins and topological edge states for fundamental antisymmetric Lamb modes in snowflakelike phononic crystal slabs. *J. Acoust. Soc. Am.* **146**, 729 (2019).

9 Long, Y., Ren, J. & Chen, H. Intrinsic spin of elastic waves. *Proc. Natl. Acad. Sci. USA* **115**, 9951-9955 (2018).

10 Sun, X.-C. & Hu, X. Topological ring-cavity laser formed by honeycomb photonic crystals. *Phys. Rev. B* **103**, 245305 (2021).

11 Yu, S.-Y. et al. Critical couplings in topological-insulator waveguide-resonator systems observed in elastic waves. *Natl. Sci. Rev.* **8**, nwaa262 (2021).
